# α-Synuclein-Dependent Calcium Entry Underlies Differential Sensitivity of Cultured SN and VTA Dopaminergic Neurons to a Parkinsonian Neurotoxin

**DOI:** 10.1523/ENEURO.0167-17.2017

**Published:** 2017-11-21

**Authors:** Ori J. Lieberman, Se Joon Choi, Ellen Kanter, Anastasia Saverchenko, Micah D. Frier, Giulia M. Fiore, Min Wu, Jyothisri Kondapalli, Enrico Zampese, D. James Surmeier, David Sulzer, Eugene V. Mosharov

**Affiliations:** 1 New York State Psychiatric Institute, Columbia University Medical Center, New York, NY 10032; 2 Department of Psychiatry, Columbia University Medical Center, New York, NY 10032; 3 Department of Neurology, Columbia University Medical Center, New York, NY 10032; 4 Department of Pharmacology, Columbia University Medical Center, New York, NY 10032; 5 Department of Physiology, Northwestern University Feinberg School of Medicine, Chicago, IL 60611

**Keywords:** α-synuclein, calcium, mitochondria, dopamine, Parkinson’s disease, neurodegeneration, MPP^+^, MPTP

## Abstract

Parkinson’s disease (PD) is a debilitating neurodegenerative disease characterized by a loss of dopaminergic neurons in the substantia nigra (SN). Although mitochondrial dysfunction and dysregulated α-synuclein (aSyn) expression are postulated to play a role in PD pathogenesis, it is still debated why neurons of the SN are targeted while neighboring dopaminergic neurons of the ventral tegmental area (VTA) are spared. Using electrochemical and imaging approaches, we investigated metabolic changes in cultured primary mouse midbrain dopaminergic neurons exposed to a parkinsonian neurotoxin, 1-methyl-4-phenylpyridinium (MPP^+^). We demonstrate that the higher level of neurotoxicity in SN than VTA neurons was due to SN neuron-specific toxin-induced increase in cytosolic dopamine (DA) and Ca^2+^, followed by an elevation of mitochondrial Ca^2+^, activation of nitric oxide synthase (NOS), and mitochondrial oxidation. The increase in cytosolic Ca^2+^ was not caused by MPP^+^-induced oxidative stress, but rather depended on the activity of both L-type calcium channels and aSyn expression, suggesting that these two established pathogenic factors in PD act in concert.

## Significance Statement

The authors investigated the mechanism of differential sensitivity of substantia nigra (SN) and ventral tegmental area (VTA) dopaminergic neurons to a mitochondrial neurotoxin 1-methyl-4-phenylpyridinium (MPP^+^). They demonstrate that α-synuclein (aSyn)- and L-type calcium channel-dependent elevation of calcium is the primary cause of mitochondrial oxidation and toxicity in SN neurons. This finding connects two disparate areas of Parkinson’s disease (PD) research and uncovers a novel interaction between the primary genetic risk factor for PD, mitochondrial dysfunction, and calcium signaling.

## Introduction

Parkinson’s disease (PD) is a debilitating neurodegenerative disorder defined by bradykinesia, postural instability, resting tremor, and muscular rigidity. Although significant symptomatic benefits can be provided to patients by treatment with levodopa, no disease modifying treatment exists for PD, indicating that a better understanding of underlying pathologic mechanisms is needed to develop improved therapy. Patients with PD demonstrate selective cell death of dopaminergic neurons in the substantia nigra (SN) with relative sparing of neighboring dopamine (DA) neurons of the ventral tegmental area (VTA). Notably, proteinaceous α-synuclein (aSyn)-positive deposits, Lewy bodies, are found in the ventral midbrain and other affected brain areas, and mutations or gene multiplication of aSyn cause autosomal dominant PD, suggesting a role for this protein in mediating disease pathology. Finally, mitochondrial dysfunction has also been proposed to underlie DA neurons degeneration as several PD genes encode proteins related to normal mitochondria homeostasis (Ryan et al., 2015). Identifying how aSyn and mitochondrial dysfunction may interact to yield selective cell death represents an attractive approach to understanding PD pathophysiology.

The environmental neurotoxin 1-methyl-4-phenyl-1,2,3,6-tetrahydropyridine (MPTP) recapitulates many pathologic features of idiopathic PD (Davis et al., 1979; Langston et al., 1983), including selective degeneration of SN dopaminergic neurons, and the dependence of this toxicity on the expression of endogenous aSyn (Dauer et al., 2002). Understanding how a putative mitochondrial poison could yield selective degeneration of SN but not VTA neurons may uncover novel approaches to develop disease-modifying therapies for PD and related neurodegenerative disorders.

MPTP is metabolized by astroglia to the active toxin, 1-methyl-4-phenylpyridinium (MPP^+^), which accumulates in DA neurons as a substrate for the DA uptake transporter (DAT; Javitch et al., 1985). Hypotheses on how MPP^+^ leads to selective SN neuron death can be subsumed into two categories. First, increased toxicity may result from a higher intracellular concentration of MPP^+^ achieved inside SN neurons. This has been credited to extraneuronal factors, such as a region-specific expression of the transporters that release MPP^+^ from astroglia (Cui et al., 2009), or increased uptake of the toxin due to higher expression of DAT in SN than VTA neurons (Shimada et al., 1992; Hurd et al., 1994; Sanghera et al., 1997; González-Hernández et al., 2004). Alternatively, SN neurons may be more sensitive to MPP^+^ due to intrinsic factors, including higher axonal arborization leading to elevated bioenergetic requirements (Pacelli et al., 2015), higher levels of cytosolic DA (DA_cyt_; Mosharov et al., 2009), or selective expression of the voltage-gated L-type calcium channel (Ca_v_1.3; Chan et al., 2007) and GirK2 potassium channels (Chung et al., 2005), activation of K-ATP channels (Liss et al., 2005) or dynamin-like protein 1 (Wang et al., 2011).

Here, we used electrochemical and imaging methods on primary mouse DA neuronal cultures to determine factors underlying the selectivity of cell death following MPP^+^ exposure. We show that, although there is higher DAT activity in SN compared to VTA DA neurons, this cannot fully account for the difference in their susceptibility to MPP^+^. In contrast, we found that the toxin selectively elevated cytosolic and mitochondrial Ca^2+^ in SN neurons, and that blocking this response decreased mitochondrial oxidation and neurotoxicity. The increase in Ca^2+^ following MPP^+^ exposure was dependent on the activities of L-type Ca^2+^ channels (LTCCs) and ryanodine receptors (RyRs), and the expression of aSyn. Overall, our data support a role for disrupted Ca^2+^ homeostasis in PD and other synucleinopathies and suggest that two well-known players of PD pathogenesis converge on the same neurotoxicity pathway.

## Materials and Methods

### Animals and neuronal cultures

Mice were housed according to the National Institutes of Health guidelines under a 12/12 h light/dark cycle with *ad libitum* access to food and water. All animal studies were reviewed and approved by the Institutional Animal Care and Use Committee. C57BL/6, DAT^IREScre^ (strain name: B6.SJL-Slc6a3^tm1.1(cre)Bkmn^/J, RRID:IMSR_JAX:006660) and Ai38 floxed GCaMP3 reporter (strain name: B6;129S-Gt(ROSA)26Sor^tm38(CAG-GCaMP3)Hze^/J, RRID:IMSR_JAX:014538) mouse lines were obtained from The Jackson Laboratory. For measurements of mitochondrial oxidation in combination with Ca^2+^ and NO probes, we used cultures from transgenic mice expressing a mitochondrially targeted ratiometric redox probe, roGFP (Dooley et al., 2004) under the control of the TH promoter (TH-mito-roGFP; Guzman et al., 2010). For DA_cyt_ measurements, we used cultures from mice that express green fluorescent protein under the control of the rat tyrosine hydroxylase promoter (TH-GFP; Sawamoto et al., 2001). SN and VTA DA neurons from postnatal day 0–2 mice of either sex were dissected, dissociated, and plated on a monolayer of rat cortical astrocytes at the plating density of ∼100,000 cells/cm^2^, as described previously (Rayport et al., 1992); experiments were conducted 10–15 d after plating.

To overexpress human wild-type (WT) aSyn, we used adeno-associated virus serotype 2 (AAV2)-aSyn under the control of chicken β-actin (CBA) promoter produced by the UNC Vector Core Facility and available from the Michael J. Fox Foundation (New York, NY). As a control, we employed AAV2-mKate2 (red fluorescence; Shcherbo et al., 2009; Caraveo et al., 2014). The viruses were used on cultures from DAT-GCaMP3 animals at a 20,000-30,000 genome copies/cell concentration, resulting in >90% infection of dopaminergic neurons. GCaMP3 fluorescence measurements were performed 5-7 d postinfection.

### Neurotoxicity assays

Cells were preincubated with various metabolic effectors for the time indicated in the text before the application of either MPP^+^ or pierecidin A (Sigma). After 48 h, immunostaining of 4% paraformaldehyde-fixed cultures was performed using mouse anti-TH antibodies (1:1000; Millipore Bioscience Research Reagents; RRID: AB_2201526) followed by secondary antibodies conjugated with Alexa Fluor 488 (1:300; Invitrogen; RRID: AB_141607). For each dish the number of immunereactive cells in 20 fields of view at 200× magnification (Plan-Neofluar 20× objective; ∼0.8 mm^2^ viewing field) was tallied and the average density was calculated (Mosharov et al., 2009). The counts were performed by an observer blind to the experimental treatments; each experimental condition was repeated at least twice with at least three dishes per condition in each of the experiments.

### Electrophysiological recordings

For whole-cell patch-clamp recording from cultured VTA and SN DA neurons, cells were kept at room temperature in Tyrode’s solution containing 119 mM NaCl, 3 mM KCl, 10 mM glucose, 2 mM CaCl_2_, 1.2 mM MgCl_2_-6 mM H_2_O, 3.3 mM HEPES, and 2.7 mM HEPES-Na^+^ salt; pH 7.2–7.4, 270 mOsm. Borosilicate glass pipettes with a tip resistance of 3–4 MΩ (G150F-4; Warner Instruments) were pulled on a P-97 Flaming-Brown micropipette puller (Sutter Instruments) and filled with 115 mM K-gluconate, 20 mM KCl, 10 mM HEPES, 2 mM MgCl_2_, 2 mM ATP-Mg, 2 mM ATP-Na_2_, and 0.3 mM GTP-Na; pH 7.25, ∼280 mOsm. Neurons were visualized under a 40× water immersion objective by fluorescence and DIC optics (BX51; Olympus). Whole-cell current clamp recording were performed with an Axopatch 700B amplifier (Molecular Devices) and digitized at 10 kHz with ITC-18 (HEKA Instruments). Data were acquired using WinWCP software (written by John Dempster, University of Strathclyde, United Kingdom). Spontaneous firing frequency was measured in cell-attached mode, then cell membrane was ruptured and holding potential adjusted to -60 mV. In each cell, membrane capacitance, input resistance and evoked action potentials were measured by injecting somatic currents from 0 to +190 pA in +10-pA increments for 1 s. Tetrodotoxin (TTX; 0.5 µM) was perfused to measure resting membrane potential and current−voltage relationship (-300 to +300 pA, +100-pA increments for 1 s). Data analysis and statistics were performed using Clampfit (Molecular Devices) and GraphPad Prism (GraphPad software). Data are presented as mean ± SEM with statistical analysis run by Mann-Whitney *t* test and two-way ANOVA.

### Measurements of DA_cyt_ by intracellular patch electrochemistry (IPE)

Measurements of neuronal DA_cyt_ in cultures from TH-GFP mice were performed as described previously (Mosharov et al., 2009). Briefly, we used an IPE electrode holder that allowed to house a polyethylene-coated 5-μm carbon fiber electrode (CFE) inside the glass patch pipette. Voltage ramps from −450 mV holding potential to +800 mV over 8.5 ms (scan rate of 295 mV/ms) were applied to the CFE at 100 ms intervals using a subroutine locally written in IGOR Pro (WaveMetrics). Subtraction voltammograms were generated, and DA concentration at the maximum of the oxidation wave was calculated using calibration curves generated for CFEs with different detection surface areas (Mosharov et al., 2003). The initial DA concentration in cellular cytosol was calculated using the cell body volume and the volume of the pipette tip estimated from photographs taken before each recording. All drugs and inhibitors were present in the bath and in the patch pipette. Because the concentration of DA_cyt_ is below the detection limits in untreated DA neurons, cultures were preincubated for 30 min with 100 μM l-DOPA. The drug was also present in the bath and in the patch pipette during the recordings, which increased the baseline oxidation current but did not otherwise interfere with DA measurements. Within each experiment, the same CFE was used for measurements from experimental and control groups of cells. IPE measurements were performed at room temperature.

### DAT activity

To estimate DAT activity, we employed 4-(4-dimethylamino)phenyl-1-methylpyridinium (APP^+^), a fluorescent analog of MPP^+^ and a DAT substrate (Karpowicz et al., 2013). Cells from WT mice were incubated with indicated concentrations of APP^+^ for 10 min, rinsed twice and imaged using 410 nm excitation and 535 nm emission filters.

As an independent approach to measure DAT-mediated uptake, SN and VTA neuronal cultures were preincubated with 10 μM pargyline and 2 μM reserpine for 30 min and then with 10 μM DA for 60 min at 37°C. Extracellular DA was then rinsed and DA_cyt_ measured by IPE as above. DAT-specific transport was derived as a difference between DA_cyt_ concentrations in the presence and in the absence of 10 μM nomifensine (added 30 min before DA). HPLC measurements performed under the same experimental conditions showed that cellular DA is stably elevated for at least 30 min after the external DA washout (data not shown).

### Fluorescent microscopy

For live cell imaging, cultures were mounted on an open perfusion chamber and superfused with recording saline (0.5 ml/min at ∼30°C). Cultures were imaged with an Olympus IX81 inverted fluorescence microscope equipped with a digitized stage (ProScan; Prior Scientific), a 63×/1.35 oil objective (Olympus), a corresponding fluorescence filter set and a 2.0 neutral density filter using a CoolSNAP HQ camera (Roper Scientific/Photometrics) and MetaMorph software (Molecular Devices). For each fluorescent reporter, 10-50 images or pairs of images were acquired at 200-ms exposure time at a frequency of 1-2 Hz. Analysis was performed with ImageJ software (NIH). Images were background corrected frame by frame by subtracting out the mean pixel values of a cell-free region or underlying glial cells depending on the location of the cell. For each time frame, fluorescence intensity was represented by the mean of averaged pixel intensities in three regions of neuronal soma, excluding the nucleus. The average of a time series represented neuron fluorescence used for statistical analysis.

For immunofluorescence, paraformaldehyde-fixed cultures were labeled at 4°C overnight with mouse anti-TH antibodies (1:1000; Millipore Bioscience Research Reagents; RRID: AB_2201526), rabbit anti ATF6 (1:500; ab37149 from ABCAM; RRID: AB_725571), or aSyn (1:100; clone 42, BD Biosciences; RRID: AB_398107), followed by Alexa Fluor fluorescent secondary antibodies (1:300; Invitrogen). Cells were imaged using 63×/1.35 oil objective (Olympus), a corresponding fluorescence filter set and a 2.0 neutral density filter using an Orca Flash 4 V3 camera (Hamamatsu) and MetaMorph software (Molecular Devices; RRID: SCR_002368). aSyn immunofluorescence was determined in the cell soma, excluding the nucleus. For the quantification of ATF6 intracellular distribution, the ratio of fluorescence intensities inside the nucleus and in the perinuclear region of the cytosol was calculated for each cell.

### Mitochondrial oxidation

For each cell from TH-mito-roGFP mice, 10 series of pairs of fluorescent images at 410 and 470 nm excitation and 535 nm emission were taken (Chroma Technology, filter set 71012). At the end of each experiments, the maximal and minimal fluorescence of roGFP was determined by a 15-min application of 2 mM dithiothreitol (DTT) to fully reduce the probe, and then 2 mM hydrogen peroxide (H_2_O_2_) to fully oxidize it. Relative oxidation of the mitochondria was calculated as previously described (Guzman et al., 2010) as 1 – ((F – F_H2O2_)/F_DTT_ – F _H2O2_)).

### Mitochondria morphology

Mitochondria morphology was analyzed as described previously (Dagda et al., 2009) using ImageJ. Briefly, epifluorescent images of TH-mito-roGFP signal were median filtered and loaded into the mitochondrial morphology plugin to obtain mitochondrial circularity data. For morphology comparisons between aSyn knock-out and WT neurons, cells were incubated with 1 µM mitotracker Red for 1 h followed by a 30-min wash in Tyrode’s saline and imaged using 560 nm excitation and 630 nm emission (Chroma Technology, filter set 49008). Epifluorescent microscope images were median filtered and the Feret diameter of mitotracker-labeled puncta were manually determined for each cell by an observer blind to genotype and treatment condition. DA neurons were identified by labeling with APP^+^.

### Mitochondrial Ca^2+^

Adeno-associated virus that expresses mitochondrial GCaMP6m (*K*_d_ = 167 nM Ca^2+^) under the control of rat TH promoter was developed as follows. The plasmid for the mitochondrial matrix targeted Ca^2+^ sensor GCaMP6m (Logan et al., 2014; Patron et al., 2014) was a generous gift from Dr. Diego De Stefani (Department of Biomedical Sciences, University of Padova, Italy). A 1558-bp 2mt-GCaMP6m insert was PCR amplified using forward (AACTTAAGCTTGGTACCGAGCTCGGATCCATG) and reverse (GAATTCTCACTTCGCTGTCATCATTTGTACAAAC) primers. The PCR fragment was subcloned into EcoR1 site of pAAV-TH-Sv40 poly A vector. The final construct was packaged into AAV serotype 9 (AAV9) by Virovek.

WT midbrain cultures (14 days in vitro) were treated with the AAV9 TH-2mt-GCaMP6m viral stock (2.41^+13^ viral particles/ml) as follows. All media, except ∼100 μl, were removed, 0.5 μl of the stock added overnight, and 2 ml of fresh media added the next day. Cells were used 4-5 d postinfection. Poststaining of midbrain cultures infected with the virus showed that >85% of 2mt-GCaMP6m-positive neurons were TH-positive (41 of 47 cells). Cells expressing 2mt-GCaMP6m were imaged at 410 and 470 nm excitation and 535 nm emission (Chroma Technology, filter set 71012). As exciting GCaMP6m at 410 nm isosbestic point led to fluorescence emission that was not Ca^2+^ dependent (Logan et al., 2014), the ratio between the excitation wavelengths of 470 and 410 nm was proportional to the Ca^2+^ concentration and independent of probe expression levels.

### Intracellular Ca^2+^

For measurements using Rhod2-AM (Invitrogen), fura-2 AM (Invitrogen), or Calcein Blue-AM (Invitrogen), we used cultures from TH-GFP and TH-mito-roGFP mice for the identification of DA neurons. Stock solution of an AM ester (1-5 μl; 1 μg/ml DMSO) was mixed with 1 μl of pluronic-F-127 solution (20% in DMSO; Invitrogen), sonicated briefly and added to the culture dish containing 2 ml of medium. While Rhod2-AM can be chemically reduced to its protonated form that should preferentially accumulate in mitochondria (Hajnóczky et al., 1995), this procedure employs a strong reducing agent, sodium borohydride (NaBH_4_), that can interfere with the effects of MPP^+^. We therefore used a regular, non-reduced form of Rhod2-AM that resulted in a mostly cytosolic and nuclear staining of DA neurons.

After rinsing the cells twice with recording saline, dishes were mounted in the perfusion chamber and imaged 30 min later at ∼30°C. Fura-2 was imaged using a filter wheel (Prior Scientific), switching between 340 and 380 nm excitation filters (emission, 510 nm; Chroma Technology, filter set 79001 with a 1.3 neutral density filter). Rhod-2 was imaged using 560 nm excitation and 630 nm emission (Chroma Technology, filter set 49008). Calcein Blue was imaged at 350 nm excitation and 460 nm emission (Chroma Technology, filter set 31000v2).

To obtain transgenic mice expressing GCaMP3 (Tian et al., 2009) in the cytoplasm of dopaminergic neurons, we crossed the Ai38 floxed GCaMP3 reporter (Zariwala et al., 2012) and DAT^IREScre^ (Bäckman et al., 2006) mouse lines. DAT^IREScre^/WT heterozygous mice were crossed with heterozygous Ai38 mice and the resulting heterozygous mice for both transgenes were identified by PCR according to protocols provided on the vendor’s website and used to produce SN and VTA cultures. Imaging of GCaMP3 was performed with 470 nm excitation and 525 nm emission (Chroma Technology, filter set 49002) at 37°C.

Comparison of the basal Ca^2+^ levels in SN and VTA neurons using three different fluorescent reporters yielded conflicting results: Rhod2 fluorescence was significantly higher in untreated SN than VTA neurons (see Results), the difference with GCaMP3 was smaller and did not reach significance, whereas fura-2 showed no difference (Dryanovski et al., 2013). A possible explanation might be the difference in the Ca^2+^ binding constants between the dyes, which is highest for Rhod2 (570 nM), intermediate for GCaMP3 (405 nM) and lowest for fura-2 (224 nM). Additionally, intracellular compartmentalization of the dyes might be different: GCaMP3 is a cytosolic protein, fura-2 has been shown to localize to other organelles (Takeuchi et al., 1989), while Rhod2 may accumulate into the mitochondria (Hajnóczky et al., 1995). Importantly, however, all three reporters demonstrated the MPP^+^-mediated increase in Ca^2+^ in SN but not in VTA dopaminergic neurons.

### Nitric oxide (NO)

To determine changes in intracellular NO levels, we used midbrain cultures from TH-mito-roGFP mice and pretreated them with a cell-permeable NO fluorescent indicator, DAR-4M-AM (10 μM, Santa Cruz Biotechnology) for 10 min at 37°C before the recordings. Cultures were then rinsed three times with recording saline, mounted in the perfusion chamber (∼30°C), and imaged 30 min later using 560 nm excitation and 630 nm emission filters.

### Data analysis

All electrochemical and optical recordings were performed on sister cultures pretreated with indicated inhibitors and MPP^+^, except Figure 4*F*, where fluorescent images were taken from the same cells. Statistical analysis was performed in Prism 4 (GraphPad Software; RRID:SCR_002798), using one-way ANOVA followed by a Tukey’s *post hoc* test for comparisons across multiple groups or two-way ANOVA with Bonferroni *post hoc* test for the paired data. In some cases, data in each experiment performed on sister cultures were normalized to values in control samples and pooled for statistical analysis. Data on all figures are represented as mean ± SEM. Details of statistical analysis for all datasets are presented in Table 1.

## Results

To address the mechanisms underlying the cell-type selective effects of MPP^+^ exposure, we used cultured primary mouse dopaminergic neurons. We have shown previously that DA neurons in these cultures exhibit intrinsic activity, including tonic and phasic firing, and a voltage sag on brief injection of strong hyperpolarizing currents (Rayport et al., 1992; Dryanovski et al., 2013) similar to those observed in the acute brain slice (Guzman et al., 2010; Tateno and Robinson, 2011; McCutcheon et al., 2012; Roeper, 2013; Avegno et al., 2016; Extended Data Fig. 1-1*A*,*B*). Using anatomic criteria, we prepared cultures from either the medial ventral midbrain (VTA) or lateral ventral midbrain (SN). We have previously shown that cultures of SN neurons contain a fraction (<25%) of calbindin-positive VTA dopaminergic neurons and vice versa (Mosharov et al., 2009). Using cultures from mice that express green fluorescent protein under the control of the tyrosine hydroxylase promoter (TH-GFP) to identify individual DA neurons, we compared basic electrophysiological properties of cultured SN and VTA neurons. Spontaneous firing frequency measured in cell-attached mode was not different between the two neuronal populations [VTA: 3.8 ± 0.3 Hz (*n* = 8); SN: 3.2 ± 1.0 Hz (*n* = 9), *p* = 0.1388 by *t* test]. Whole-cell current-clamp recordings showed slightly higher firing frequency on current injections in SN neurons, but no differences between these two DA neuron populations in rheobase, IV curve, resting membrane potential, input resistance or membrane capacitance (Extended Data Fig. 1-1*C-H*). Overall, primary DA neurons in culture express biochemical markers and demonstrate morphologic and electrophysiological properties that are comparable to those in acute slice preparations (Branch et al., 2014; Dufour et al., 2014). Thus, this system, which excludes extraneuronal contributors to MPTP toxicity, is sufficiently robust to study intraneuronal factors that lead to MPP^+^-mediated cell death. To reduce variability and minimize the potential effect of neuronal dedifferentiation, experiments were performed on sister cultures of SN and VTA neurons with the expression of dopaminergic markers (TH or DAT) confirmed in live or postfixed cells.

**Figure 1.**
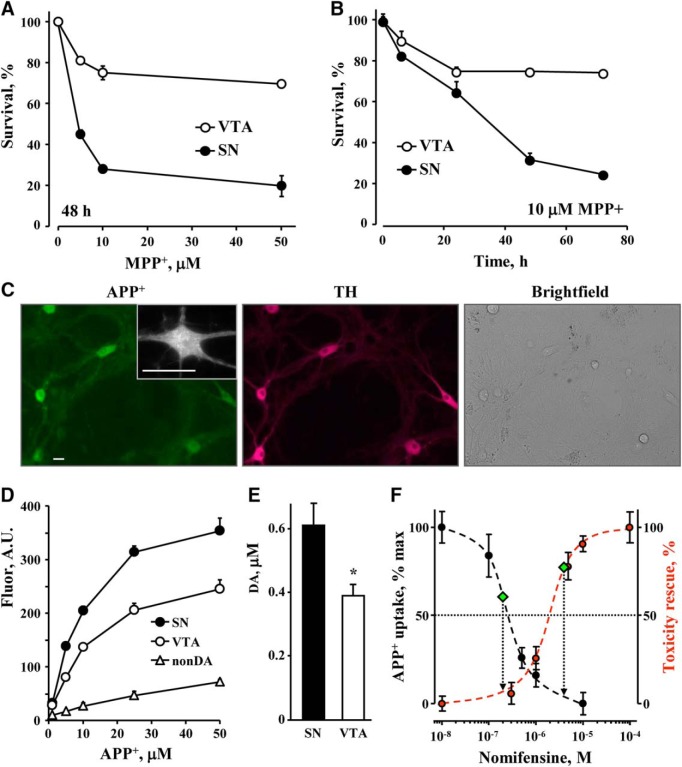
MPP^+^-induced toxicity in cultured SN and VTA DA neurons. ***A***, Neurotoxicity following a 2-d exposure of primary cultures of mouse WT SN and VTA neurons to different concentrations of MPP^+^. DA neurons were tallied following fixation and immunostaining for TH; *p* < 0.001 by two-way ANOVA (*n* = 9-23 dishes in each group from 12 independent experiments). Basal electrophysiological characteristics of cultured mouse midbrain neurons are shown in Extended Data Figure 1-1. MPP^+^-mediated toxicity in neuronal cultures from different mouse strains is shown in Extended Data Figure 1-2. ***B***, Time course of toxicity in cultures treated with 10 µM MPP^+^; *p* < 0.001 by two-way ANOVA (*n* = 3-6 dishes from two independent experiments). ***C***, Representative images of midbrain cultures treated with 5 µM APP^+^ for 10 min and then fixed and stained for TH. All APP^+^-positive cells were TH-positive, whereas 95% of TH-positive neurons were APP^+^-positive (*n* = 17). Inset demonstrates punctate APP^+^ staining in neuronal somas and processes. Scale bar: 10 µm. ***D***, Concentration dependence of APP^+^ uptake into SN and VTA neurons, as well as non-DA cell in the same cultures. Lineweaver-Burk plot of the data are shown in Extended Data Figure 1-3. ***E***, Average nomifensine-sensitive (10 µM; 30 min) part of DA uptake into SN and VTA neurons pretreated for 30 min with MAO and VMAT inhibitors pargyline (10 µM) and reserpine (2 µM), correspondingly, and then treated with 10 µM DA for 1 h (*p* < 0.05 by *t* test). DA_cyt_ concentrations were determined by IPE in the cyclic voltammetric mode of detection (Extended Data Fig. 1-4). ***F***, Dependence of APP^+^ uptake (left axis) and survival of MPP^+^-treated SN neurons (right axis) on nomifensine concentration. For DAT activity assay, cells were preincubated with nomifensine for 30 min, and then exposed to 10 µM APP^+^ for another 10 min. Fluorescence intensity of the cell bodies was quantified, background subtracted and normalized to that in nomifensine-untreated SN neurons. The IC_50_ of the DAT inhibition is ∼250 nM nomifensine. Toxicity was measured following cell incubation with indicated concentrations of nomifensine and 10 µM MPP^+^. Zero toxicity rescue corresponds to the levels of MPP^+^ toxicity in the absence of nomifensine, and 100% to that in untreated SN neuronal cultures. The IC_50_ of the nomifensine-mediated rescue of SN neurons is ∼2 µM. Left and right diamonds represent DAT activity and toxicity in VTA neurons, respectively.

Exposure of cultured ventral midbrain neurons to a range of MPP^+^ concentrations confirmed that, as *in vivo*, SN neurons were far more sensitive to the toxin than VTA neurons (Fig. 1*A*; Extended Data Fig. 1-2), similar to other reports (Pacelli et al., 2015). Significant differences were also observed in the time course of toxicity, with most cell death occurring on the first day of exposure in VTA neurons and over 3 d in the SN cultures (Fig. 1*B*).

10.1523/ENEURO.0167-17.2017.f1-1 Figure 1-1 Electrophysiological properties of cultured primary DA neurons from SN and VTA. ***A***, Examples of cell-attached recordings of spontaneous activity in ventral midbrain cultures from TH-GFP mice. ***B***, Change in the firing frequency of cultured DA neurons following treatment with a cocktail of postsynaptic neurotransmission blockers AP5 (50 μM, NMDA receptor antagonist), CNQX (10 μM, AMPA receptor antagonist), and bicuculine (25 μM, GABAA receptor antagonist). Phasic firing was reduced in the presence of blockers of GABAergic and glutamatergic synaptic activity as seen by a loss of events with high instantaneous frequency. ***C***, Difference in the evoked action potentials frequency between VTA and SN neurons (*p* < 0.01, two-way ANOVA). Average action potential for each 1-s somatic step currents is plotted as a function of injected current amplitude (+10 to +190 pA, 10-pA increments). ***D***, There was no significant difference in rheobase (minimum current required to elicit first action potential) between DA neurons in VTA (26.2 ± 6.8 pA, *n* = 13) and SN (21.8 ± 6.6 pA, *n* = 11) by *t* test. ***E***, No difference in voltage response to somatic current injection (-300 to +300 pA, +100-pA increments) of DA neurons in VTA and SN in the presence of 1 µM TTX (*p* > 0.05, two-way ANOVA). ***F***, No difference in average resting membrane potential between DA neurons in VTA (-43.8 ± 2.3 mV, *n* = 15) and SN (-42.1 ± 2.5 mV, *n* = 12) by *t* test. ***G***, No difference in input resistance between VTA (676 ± 74.2 MΩ, *n* = 14) and SN (650 ± 83.3 MΩ, *n* = 12) neurons. ***H***, No difference in membrane capacitance between VTA (59.8 ± 16.8 pF, *n* = 14) and SN (50 ± 7.70 pF, *n* = 12) neurons. Download Figure 1-1, TIF file.

**Figure 2.**
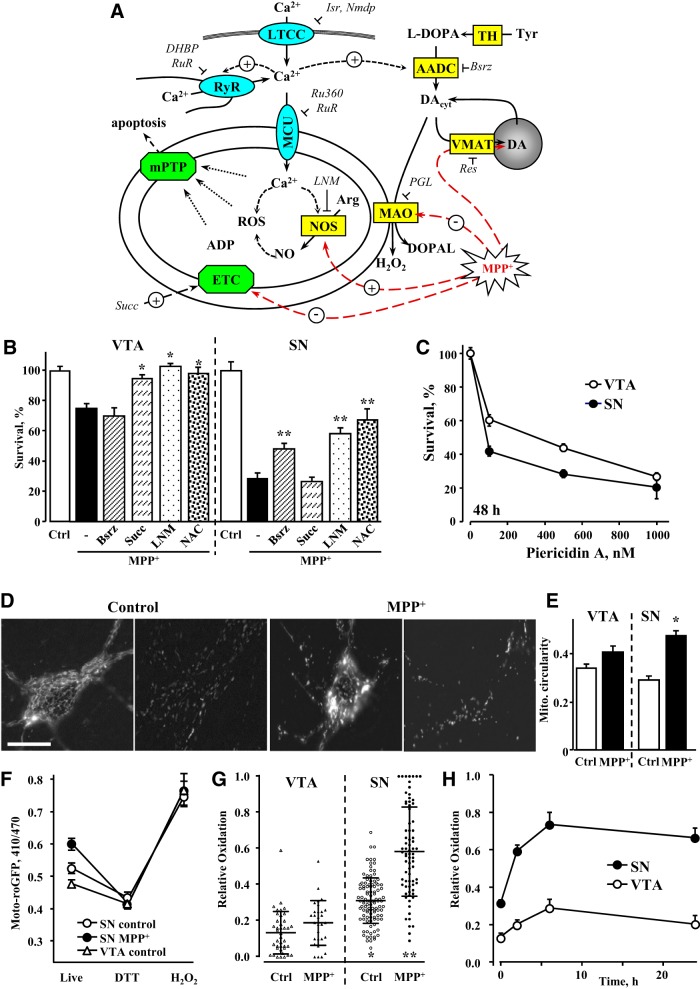
Difference in mechanisms of MPP^+^-induced toxicity in SN and VTA neurons. ***A***, Schematics of possible MPP^+^ toxicity pathways. Abbreviations, concentrations of drugs, and preincubation times used for pharmacological analysis: AADC, aromatic l-amino acid decarboxylase; Arg, arginine; ADP, adenosine diphosphate; Bsrz, benserazide (10 µM, 48 h); Ca_v_1.3, voltage-gated L-type calcium channel; DHBP, 1,1'-diheptyl-4,4'-bipyridinium dibromide (100 µM, 30 min); DOPAL, 3,4-dihydroxyphenylacetaldehyde; Isr, isradipine (5 µM, 30 min); l-DOPA, l-3,4-dihydroxyphenylalanine (100 µM, 30 min); LNM, NG-nitro-l-arginine methyl ester (l-NAME; 100 µM, 1 h); mPTP, mitochondrial permeability transition pore; Nmdp, nimodipine (5 µM, 30 min); Res, reserpine (10 µM, 24 h); ROS, reactive oxygen species; Ru360 (10 µM, 30 min); RuR, ruthenium red (20 µM, 24 h); Succ, succinate (Complex II substrate; 1 mM, 1 h); TH, tyrosine hydroxylase. Channels are shown in turquoise, enzymes and transporters in yellow and protein complexes in green. ***B***, Pharmacological analysis of known mechanisms of MPP^+^ toxicity in VTA and SN DA neurons. Cultures were treated with various pharmacological agents as described above followed by 10 µM MPP^+^; surviving TH-positive neurons were tallied 48 h later. None of the compounds were neurotoxic when applied without MPP^+^; *p* < 0.05 from MPP^+^ (*) or both MPP^+^ and control (**) by one-way ANOVA with Tukey’s *post hoc* test (*n* = 6-16 for VTA and 7-17 for SN dishes from 12 independent experiments). ***C***, Dependence of survival of SN and VTA DA neurons on the concentration of mitochondrial Complex I inhibitor pierecidin A. Following 2 d of exposure, neurons were fixed with paraformaldehyde, immunostained for TH and tallied; *p* < 0.001 by two-way ANOVA (*n* = 5-16 dishes in each group from six independent experiments). ***D***, Representative images of TH-mito-roGFP SN neuronal cell bodies (left) and axons (right) before and after 2 h of treatment with 50 µM MPP^+^. Scale bar: 5 µm. ***E***, Changes in mitochondria circularity in SN and VTA neurons treated with 50 µM MPP^+^ for 2 h; *p < 0.05 from untreated cells by one-way ANOVA with Tukey’s *post hoc* test (*n* = 14-22 cells from two independent experiments). ***F***, Average roGFP 410/470 fluorescence ratios in the somas of neurons either before (live) or after treatment with 1 mM DTT (middle) followed by 2 mM H_2_O_2_ (right; *n* = 18-21 cells from three independent experiments). ***G***, Relative oxidation of VTA and SN neurons before and after treatment with 50 µM MPP^+^ for 2 h; *p* < 0.05 from control VTA (*) or control SN (**) by one-way ANOVA with Tukey’s *post hoc* test (*n* = 29-106 cells from 14 independent experiments). Horizontal bars represent means and SDs. ***H***, Time-dependent changes in mitochondrial oxidation in SN and VTA neurons treated with 50 µM MPP^+^; *p* < 0.001 by two-way ANOVA (*n* = 22-70 cells from 11 independent experiments). Neurotoxin was added at time 0.

10.1523/ENEURO.0167-17.2017.f1-2 Figure 1-2 Dose dependence of MPP^+^-mediated toxicity in SN (***A***) and VTA (***B***) DA neuronal cultures from different mouse strains. Cell were incubated with the toxin for 2 d followed by fixation and immunostaining for TH; *n* = 5-23 dishes from six independent experiments. Download Figure 1-2, TIF file.

**Figure 3.**
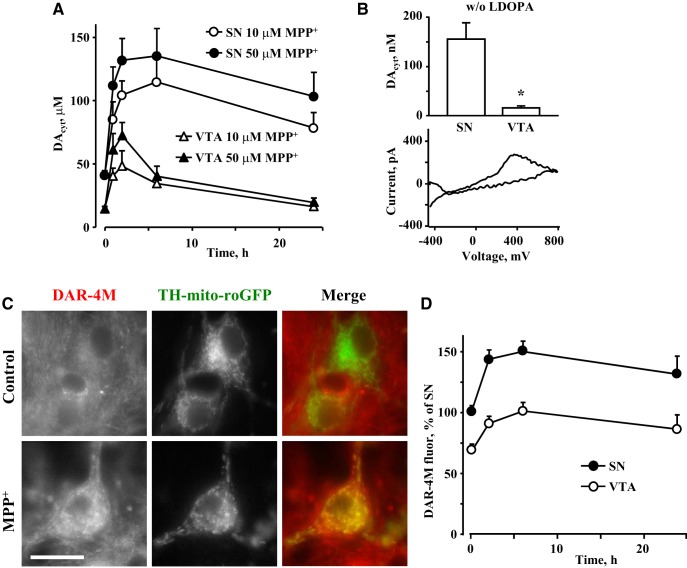
Effect of MPP^+^ on DA_cyt_ and NO. ***A***, Time course of changes in DA_cyt_ following treatment of SN or VTA neurons with 10 or 50 µM MPP^+^. All curves are statistically different from each other by two-way ANOVA (*p* < 0.01; *n* = 6-34 cells from eight independent experiments). Cells were pretreated with 100 µM l-DOPA for 30 min before the recordings. ***B***, Average DA_cyt_ concentrations (top) and representative voltammogram (bottom) in SN and VTA neurons treated with 50 µM MPP^+^ for 2 h. No l-DOPA was added before IPE measurements. Detection threshold of IPE in CV mode is ∼50 nM; *p < 0.01 by *t* test (*n* = 6 and 8 cells from two independent experiments). ***C***, Representative images of control and MPP^+^ (50 µM for 2 h)-treated SN neurons from TH-mito-roGFP mice stained with live NO indicator DAR-4M-AM. Scale bar: 10 µm. ***D***, Time-dependent changes in NO concentration in SN and VTA neurons treated with 50 µM MPP^+^. DAR-4M-AM was added during the last 10 min of MPP^+^ exposure; *p* < 0.001 by two-way ANOVA (*n* = 24-71 cells from seven independent experiments).

10.1523/ENEURO.0167-17.2017.f1-3 Figure 1-3 Following subtraction of non-specific APP^+^ staining (Fig. 1*D*), Lineweaver-Burk plot demonstrates higher V_max_ (396.6 ± 24.4 vs 237.6 ± 8.4 A.U.; *p* < 0.001 by *t* test) but similar K_0.5_ (16.9 ± 5.5 vs 15.9 ± 5 μM; n.s.) of APP^+^ uptake in SN compared to VTA neurons. Download Figure 1-3, TIF file.

**Figure 4.**
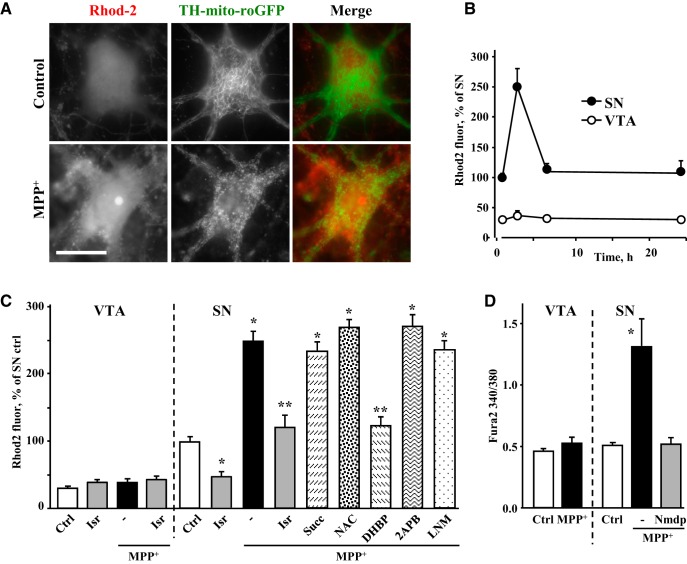
MPP^+^-induced changes in neuronal Ca^2+^. ***A***, Representative images of SN neurons from TH-mito-roGFP mice exposed to 50 µM MPP^+^ for 2 h and stained with live Ca^2+^ indicator Rhod2-AM. Scale bar: 10 µm. No difference in intracellular fluorescence of Calcein Blue was observed between SN and VTA neurons (Extended Data Fig. 4-1). ***B***, Time-dependent changes in Ca^2+^ signal in SN and VTA neurons treated with 50 µM MPP^+^. Rhod2-AM was always added during the last 30 min of MPP^+^ exposure; *p* < 0.001 by two-way ANOVA (*n* = 9-51 cells from four independent experiments). ***C***, Effect of LTCC inhibitor isradipine (5 µM), Complex II substrate succinate (1 mM), NOS inhibitor l-NAME (100 µM), RyR blocker DHBP (100 µM), or IP_3_R antagonist 2-APB (50 µM) on MPP^+^-induced Ca^2+^ elevation in SN and VTA DA neurons. Cultures were preincubated with inhibitors for 30-60 min (Fig. 2, legend) and then exposed to 50 µM MPP^+^ for 2 h; *p* < 0.01 from corresponding control (*; white bars) or MPP^+^ (**) by one-way ANOVA with Tukey’s *post hoc* test (*n* = 11-100 cells from 14 independent experiments). ***D***, Fluorescence intensity ratios at 340 and 380 nm excitation and 510 nm emission of fura-2 AM-treated SN and VTA neurons from TH-GFP mice exposed to 50 µM MPP^+^ for 2 h. LTCC inhibitor nimodipine (5 µM) was added 30 min before the toxin; *p < 0.05 from all other groups by one-way ANOVA with Tukey’s *post hoc* test (*n* = 37-68 cells from three independent experiments).

10.1523/ENEURO.0167-17.2017.f1-4 Figure 1-4 IPE setup was used to measure DA_cyt_ concentration in neurons pretreated with 100 μM l-DOPA or 10 μM DA. CFE controlled by the first amplifier (Amp 1) was used in cyclic voltammetric mode or detection. The second amplifier (Amp 2) was employed to monitor the seal formation between the cell and the glass pipette. Download Figure 1-4, TIF file.

### DAT activity in SN and VTA neurons

Why are SN neurons more susceptible to MPP^+^? To investigate whether increased intracellular accumulation of the toxin is responsible, we compared DAT activity between individual SN and VTA neurons using optical and electrochemical approaches. First, we used a recently characterized fluorescent structural analog of the toxin, APP^+^, that selectively accumulates in dopaminergic cells via DAT (Fig. 1*C*), where it co-localizes with mitochondrial markers (Karpowicz et al., 2013). Analysis of APP^+^ uptake (total cell body fluorescence intensity) revealed that SN neurons indeed possessed ∼40% higher DAT activity than VTA neurons (396.6 ± 24.4 vs 237.6 ± 8.4 A.U.; *p* < 0.001 by *t* test), but a similar K_0.5_ of the uptake (16.9 ± 5.5 vs 15.9 ± 5 μM; n.s.; Fig. 1*D*; Extended Data Fig. 1-3).

As an independent approach to compare DAT function, we employed IPE. In this technique, a CFE is positioned inside a glass patch pipette (Extended Data Fig. 1-4). After a high resistance seal is achieved, the cellular membrane is ruptured and diffusing cytosolic metabolites produce a wave of oxidation at the electrode (Mosharov et al., 2003). To measure DA_cyt_, we used IPE in cyclic voltammetric mode, which provides a means to distinguish oxidizable compounds based on their redox profile. We preincubated cells for 1 h with 10 μM DA in the presence of the monoamine oxidase (MAO) and vesicular monoamine transporter 2 (VMAT) blockers pargyline and reserpine to prevent intracellular DA breakdown and vesicular uptake, respectively. We then measured the concentration of neuronal DA_cyt_ in the presence or absence of the competitive DAT inhibitor, nomifensine. Comparison of the nomifensine-sensitive portion of intracellular DA accumulation confirmed that SN neurons exhibit ∼40% higher DAT activity (Fig. 1*E*).

To further investigate the relationship between DAT activity and MPP^+^-mediated toxicity, we compared APP^+^ uptake and neuroprotection in SN neurons pretreated with a range of nomifensine concentrations to partially block DAT. A far lower concentration of nomifensine was required to block APP^+^ uptake (IC_50_ of ∼250 nM) than to achieve a half-maximal effect on cell survival (∼2 µM; Fig. 1*F*). In fact, the nomifensine concentration that decreased DAT activity in SN neurons to the level of VTA neurons decreased toxicity by only ∼10%. Conversely, to achieve the same level of toxicity as in VTA neurons, DAT uptake needed to be blocked by ∼90% in SN neurons (Fig. 1*F*, diamonds), suggesting that while DAT is essential for MPP^+^ toxicity, additional intrinsic factors play a major role in differential sensitivity of SN and VTA DA neurons to MPP^+^.

### Different mechanisms of MPP^+^ toxicity in SN and VTA neurons

MPP^+^ has been reported to target multiple intracellular systems, particularly Complex I of the mitochondrial electron transport chain (ETC), NO synthesis and DA homeostasis (Fig. 2*A*), although the role of these pathways is debated (Hasbani et al., 2005; Kim et al., 2015). Interestingly, we found that the contribution from each of the pathways to toxicity was remarkably different between SN and VTA DA neurons (Fig. 2*B*). Whereas depletion of intracellular DA with the aromatic l-amino acid decarboxylase (AADC) inhibitor, benserazide (Bsrz; 10 μM; 48-h preincubation), provided partial protection of SN neurons, it had no effect on the survival of VTA neurons treated with MPP^+^. In contrast, succinate (1 mM; 30-min preincubation), a Complex II substrate that bypasses MPP^+^-mediated inhibition of Complex I (Lotharius and O'Malley, 2000), was ineffective at rescuing SN neurons, but prevented the death of VTA neurons. The NO synthase (NOS) blocker, l-NAME (100 μM; 1-h preincubation), protected both SN and VTA neurons, albeit with different efficiency. This demonstrates that the different levels of MPP^+^-induced toxicity between SN and VTA neurons arise from different toxicity mechanisms engaged by MPP^+^. To determine if sensitivity to Complex I inhibition might contribute to the differential toxicity observed between SN and VTA cultures, we examined the effect of a highly selective and potent Complex I inhibitor, piericidin A (Choi et al., 2011). We found that toxicity was indeed higher in SN than VTA neurons, although the difference was much smaller than for MPP^+^ (Fig. 2*C*). Thus, at least a portion of the different responses of the two cell types to stress can be attributed to elevated sensitivity of SN neurons to mitochondrial ETC blockade.

Consistent with previously reported MPP^+^-induced cellular oxidative stress (Lotharius and O'Malley, 2000), we found that the glutathione precursor N-acetyl cysteine (200 μM; 2-h preincubation) rescued both SN and VTA neurons from MPP^+^ (Fig. 2*B*). To further determine the effects of the toxin on mitochondrial oxidation, we used neuronal cultures from mice that express a mitochondria-targeted redox sensitive GFP under the control of the tyrosine hydroxylase promoter (TH-mito-roGFP; Guzman et al., 2010). While untreated cells displayed a mesh-like mitochondrial network in cell bodies and elongated mitochondria in processes, MPP^+^ exposure for 2 h induced the formation of rounded mitochondria in the soma and axons, indicative of mitochondrial stress **(** Fig. 2*D*,*E*). Mito-roGFP provides ratiometric measurements of mitochondrial oxidation that are independent of the protein's expression levels. As expected, 410/470 nm fluorescence ratios of completely reduced or oxidized roGFP were the same in SN and VTA neurons, however, the signal differed significantly in living cells (Fig. 2*F*). Furthermore, after MPP^+^ treatment, roGFP oxidation was higher (Fig. 2*G*) and lasted longer (Fig. 2*H*) in SN neurons, revealing the presence of cell type-specific mitochondrial oxidative stress.

As inhibition of DA synthesis was more effective at rescuing SN neurons, we investigated if MPP^+^-induced changes in DA homeostasis may underlie higher sensitivity of these cells to the toxin. Using cultures from TH-GFP mice, we measured DA_cyt_ using IPE in CV mode (Mosharov et al., 2003). Cultures were pretreated with the DA precursor, l-DOPA (100 µM, 30 min), to increase DA_cyt_ to levels detectable by IPE, and the time course of changes in DA_cyt_ was determined in cells exposed to 10 or 50 µM MPP^+^. We found significantly higher DA_cyt_ in SN neurons at both neurotoxin concentrations (Fig. 3*A*). To confirm that differences in DA_cyt_ did not require l-DOPA, we also measured DA_cyt_ in l-DOPA-untreated neurons. Although the levels of DA_cyt_ were much lower, MPP^+^ still induced a far greater increase in DA_cyt_ in SN compared to VTA neurons (Fig. 3*B*).

Activation of NOS and generation of peroxynitrite have been implicated in mediating MPP^+^ toxicity (Schulz et al., 1995; Przedborski et al., 1996). We, therefore, determined the levels of neuronal NO using the fluorescent reporter DAR-4M-AM, which revealed significantly higher NO levels in SN than VTA DA neurons both before and after toxin exposure (Fig. 3*C*,*D*).

### Upregulation of Ca^2+^ in SN neurons exposed to MPP^+^

Higher levels of DA_cyt_ in SN neurons may result from Ca^2+^-mediated upregulation of DA synthesis via AADC (Mosharov et al., 2009). Similarly, neuronal NOS (nNOS) is regulated by Ca^2+^ via calmodulin-mediated phosphorylation (Förstermann et al., 1991; Sanchez-Padilla et al., 2014). To examine whether changes in DA_cyt_ and NO in MPP^+^-treated neurons were driven by increased Ca^2+^ levels, we employed several fluorescent Ca^2+^ reporters.

Comparison of the accumulation of Calcein Blue-acetoxymethyl (AM), a dye sensitive to Ca^2+^ only at highly alkaline pH (Huitink et al., 1974), confirmed that AM modified dye uptake and esterase activity were similar in SN and VTA neurons (Extended Data Fig. 4-1). In contrast, fluorescence intensity of the Ca^2+^-sensitive probe Rhod-2 increased sharply in SN neurons treated with MPP^+^ for 2 h, returning to pretreatment levels after 6 h of exposure (Fig. 4*A*,*B*). Importantly, both basal and maximal Ca^2+^ concentrations were higher in SN than VTA neurons and were sensitive to dihydropyridine treatment (Fig. 4*C*), suggesting that LTCC are critical mediators of these differences. A similar increase in free Ca^2+^ in SN neurons exposed to MPP^+^ and its blockade by an LTCC inhibitor, nimodipine, were observed with the ratiometric probe fura-2 (Fig. 4*D*), although this dye showed no difference in the basal Ca^2+^ levels in SN and VTA neurons (see Methods). In contrast, restoring ETC function with succinate or alleviating cellular oxidative stress with NAC did not counter the toxin-induced elevation of Ca^2+^ (Fig. 4*C*). For further analysis, we used 10 µM MPP^+^ for neurotoxicity assays (2 d of exposure) and 50 µM MPP^+^ for acute metabolic assays (2 h), as the higher MPP^+^ concentration induced a slightly more robust response in DA_cyt_ (Fig. 3*A*) and Ca^2+^ (201 ± 15% (*n* = 47) increase in Ca^2+^ for 10 µM versus 246 ± 13% (*n* = 100) for 50 µM relative to untreated SN neurons).

10.1523/ENEURO.0167-17.2017.f4-1 Figure 4-1 Calcein Blue fluorescence intensity in the somas of SN and VTA neurons. Groups are not significantly different by *t* test (*n* = 32 cells in each group from two independent experiments). Download Figure 4-1, TIF file.

**Figure 5.**
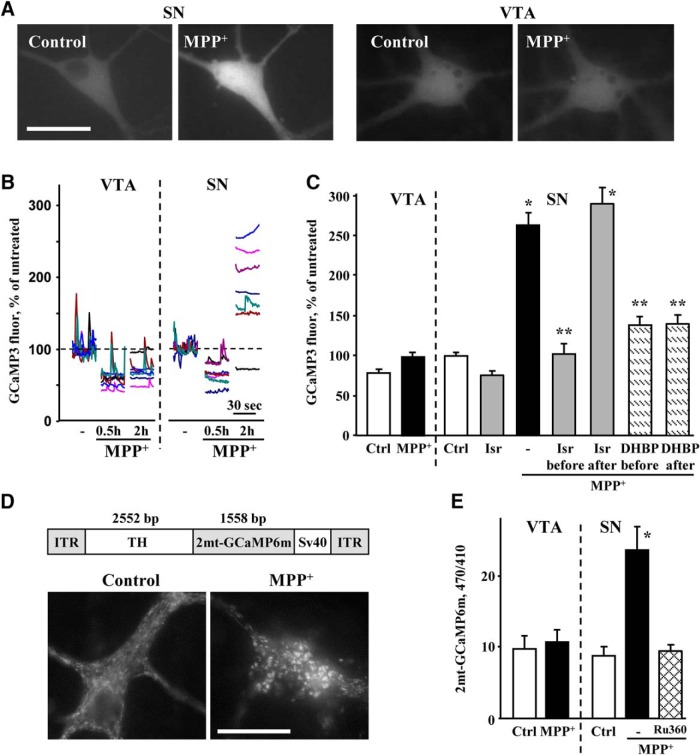
Changes in cytosolic and mitochondrial Ca^2+^. ***A***, Representative live images of SN and VTA neurons from DAT-GCaMP3 mice exposed to 50 µM MPP^+^ for 2 h. Scale bar: 10 µm. ***B***, Examples of live GCaMP3 recordings from MPP^+^-treated SN and VTA neurons. At 0, 30, and 120 min of toxin exposure, a series of 50 images at 2 Hz frequency was taken from the same cells. See Extended Data Figure 5-1*A* for an example of untreated cells and Extended Data Figure 5-1*B* for the analysis of the SD of each 30-s recording. Recordings were done at 37°C. ***C***, Changes in GCaMP3 fluorescence intensity in the cell bodies of VTA and SN neurons exposed to MPP^+^ (50 µM, 2 h) in the presence or in the absence of 5 µM isradipine or 100 µM DHBP (see Extended Data Fig. 5-1*C* for the effect of isradipine in VTA DA neurons). The blockers were added either 30 min before MPP^+^ or during the last 30 min of toxin exposure; *p* < 0.01 from control (*) or MPP^+^ (**) by one-way ANOVA with Tukey’s *post hoc* test (*n* = 20-75 cells from eight independent experiments). Extended Data Figure 5-2 shows analysis of Rhod2 fluorescence in SN neurons treated with isradipine, DHBP, and MPP^+^ the same way. ***D***, Schematics of the TH-2mt-GCaMP6m vector and representative images of untreated and MPP^+^-treated SN neurons infected with AAV9-TH-2mt-GCaMP6m. ITR, inverted terminal repeat; TH, rat tyrosine hydroxylase promoter. ***E***, Ratio of background-subtracted fluorescence intensities (410 and 470 nm excitation, 535 nm emission) of the mitochondria in SN and VTA neurons expressing 2mt-GCaMP6m; *p < 0.01 from all other groups by one-way ANOVA with Tukey’s *post hoc* test (*n* = 18-29 cells from three independent experiments).

To monitor cytosolic Ca^2+^ alterations in the same neurons before and after toxin exposure, we employed midbrain cultures from DAT-GCaMP3 mice (Fig. 5*A*). Cultured DA neurons often show Ca^2+^ transients that can be observed for several hours under our imaging conditions (Extended Data Fig. 5-1*A*). After 30 min of MPP^+^ exposure, the frequency of transients and basal Ca^2+^ levels decreased in both SN and VTA neurons (Fig. 5*B*; Extended Data Fig. 5-1*B*). In contrast to VTA neurons where GCaMP3 fluorescence did not change after the initial decrease, Ca^2+^ levels increased markedly in SN neurons treated with MPP^+^ for 2 h, although the response varied between cells. Consistent with the Rhod2 data, pretreatment with isradipine prevented MPP^+^-induced Ca^2+^ elevation in SN (Fig. 5*C*), but not VTA (Extended Data Fig. 5-1*C*) DA neurons.

10.1523/ENEURO.0167-17.2017.f5-1 Figure 5-1 ***A***, Examples of live GCaMP3 recordings in untreated SN neurons at 30 and 120 min of incubation. At each time point, a series of 50 images at 2-Hz frequency was taken from the same cells. Recordings were done at 37°C. ***B***, SD s of live recordings from SN and VTA neurons from DAT-GCaMP3 mice. At 0, 30, and 120 min of MPP^+^ exposure, a series of 50 images at 2-Hz sampling frequency was taken and SD calculated (see examples in Fig. 4*F*). MPP^+^ decreased activity-dependent Ca^2+^ spiking in both neuronal populations, with stronger effect in SN neurons. Groups are significantly different by two-way ANOVA (*p* < 0.05; *n* = 8-37 cells in each group from four independent experiments). ***C***, Average GCaMP3 fluorescence in VTA neurons treated with 50 μM MPP^+^ for 2 h in the presence and in the absence of 5 μM isradipine. None of the groups are significantly different from each other by one-way ANOVA with Tukey’s *post hoc* test (*n* = 24-57 cells from seven independent experiments). Download Figure 5-1, TIF file.

10.1523/ENEURO.0167-17.2017.f5-2 Figure 5-2 Rhod2 fluorescence intensity in the somas of SN neurons treated with 50 μM MPP^+^ for 2 h in the presence and the absence of 5 μM isradipine or 100 μM DHBP. L-type channel and RyR blockers were added either 30 min before MPP^+^ or during the last 30 min of toxin exposure. Ca^2+^ sensor Rhod2-AM was always added 30 min before the measurements of Rhod2 fluorescence; *p* < 0.05 from untreated group (*) or from MPP+ group (**) by one-way ANOVA with Tukey’s *post hoc* test (*n* = 14-100 cells in each group from seven independent experiments). Download Figure 5-2, TIF file.

Because of a reported interaction between LTCC and RyR in the brain (Chavis et al., 1996; Sanchez-Padilla et al., 2014), we investigated whether the latter were involved in mediating the increase in Ca^2+^ following MPP^+^ exposure. We inhibited LTCC or RyR either before MPP^+^ treatment or during the last 30 min of a 2-h MPP^+^ exposure. While preincubation with isradipine or an antagonist of RyR, 1,1′-diheptyl-4,4′-bipyridinium dibromide (DHBP), both prevented MPP^+^-induced elevation of Ca^2+^, delayed treatment was only effective when RyR but not when LTCC were inhibited (Fig. 5*C*). Similar responses were observed with Rhod2 (Extended Data Fig. 5-2). These data suggest that while LTCC are necessary to initiate the MPP^+^-mediated increase in Ca^2+^, they are not required once RyR are activated. In contrast, inhibition of IP_3_ receptors had no effect on Ca^2+^ levels in MPP^+^-treated SN neurons (Fig. 4*C*).

To determine the source of Ca^2+^ surge in MPP^+^-treated neurons, we employed an adeno-associated virus that expressed a mitochondria-targeted GCaMP6m under the control of rat TH promoter, TH-2mt-GCaMP6m (Logan et al., 2014). Similar to other fluorescent probes, normalized fluorescence intensity (470/410 nm excitation ratio) of 2mt-GCaMP6m increased in SN but not VTA neurons exposed to MPP^+^ (Fig. 5*D*). Importantly, this effect was blocked by an inhibitor of the mitochondrial calcium uniporter (MCU), Ru360 (Fig. 5*E*), suggesting that mitochondria were not the initial source of cytoplasmic Ca^2+^ increase, but rather accumulated it in response to changes in Ca^2+^ conductance at the plasma membrane or the endoplasmic reticulum (ER).

### Regulation of DA_cyt_ and NO by Ca^2+^

**Figure 6.**
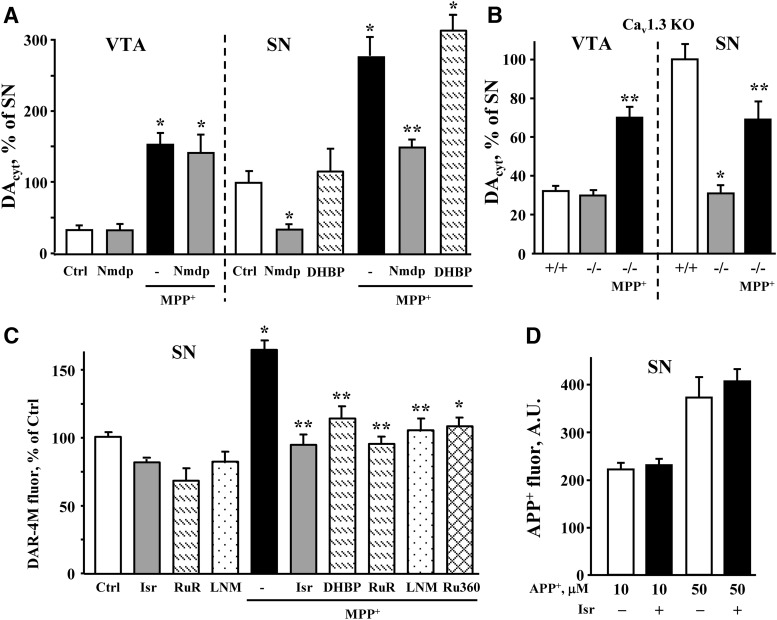
Dependence of DA_cyt_ and NO on intracellular Ca^2+^ levels. ***A***, Changes in DA_cyt_ in SN and VTA neurons exposed to 50 µM MPP^+^ for 2 h in the presence and in the absence of nimodipine (5 µM) or DHBP (100 µM) added 30 min before MPP^+^; *p* < 0.01 from corresponding control (*) or MPP^+^-treated (**) neurons by one-way ANOVA with Tukey’s *post hoc* test (*n* = 9-28 cells from four independent experiments). Incubations with all drugs were done at 37°C, followed by imaging at RT. Dependence of DA_cyt_ on nimodipine concentration in naïve SN cultures is shown of Extended Data Figure 6-1. ***B***, DA_cyt_ levels in SN and VTA neurons from WT (+/+) and Ca_v_1.3 knock out (-/-) mice; *p* < 0.01 from corresponding +/+ (*) or both +/+ and -/- groups (**) by one-way ANOVA with Tukey’s *post hoc* test (*n* = 20-64 cells from seven independent experiments). ***C***, Normalized fluorescence intensity of NO indicator DAR-4M in SN neurons pretreated with indicated inhibitors and then exposed to 50 µM MPP^+^ for 2 h; *p* < 0.001 from control (*) or MPP^+^ (**) groups by one-way ANOVA with Tukey’s *post hoc* test (*n* = 15-80 cells from 14 independent experiments). Quantification of DAR-4M fluorescence in VTA neurons treated with some of the same drugs is shown in Extended Data Figure 6-2. ***D***, Fluorescence intensity in the somas of SN and VTA neurons treated with 10 or 50 µM APP^+^ for 10 min in the presence and the absence of 5 µM isradipine (30-min preincubation). *N* = 16-20 cells.

To confirm that increased DA_cyt_ and NO in MPP^+^-treated SN neurons result from higher Ca^2+^ levels in these cells, we measured these metabolites following pharmacological blockade of LTCC with dihydropyridines. Treatment of naïve SN cultures with nimodipine decreased DA_cyt_ in a concentration-dependent manner (Extended Data Fig. 6-1), and the IC_50_ of this effect (∼2.5 µM) was similar to that of the inhibition of the Ca_v_1.3 channel (Mercuri et al., 1994). Furthermore, nimodipine (5 µM, 30-min preincubation) abolished the difference in DA_cyt_ between SN and VTA neurons both in the presence and in the absence of MPP^+^ (Fig. 6*A*). Similarly, when IPE measurements were performed in cultures from the Ca_v_1.3 deficient mice, there was no difference in DA_cyt_ levels in SN and VTA neurons, either in the presence or in the absence of the toxin (Fig. 6*B*). In contrast, the RyR blocker DHBP had no effect on DA_cyt_ in MPP^+^-treated SN neurons (Fig. 6*A*).

10.1523/ENEURO.0167-17.2017.f6-1 Figure 6-1 Dependence of DA_cyt_ in SN neurons on the concentration of LTCC blocker nimodipine (30-min pretreatment); *p < 0.05 from untreated group by one-way ANOVA with Tukey’s *post hoc* test (*n* = 9-33 cells from six independent experiments). Download Figure 6-1, TIF file.

10.1523/ENEURO.0167-17.2017.f6-2 Figure 6-2 Measurements of NO levels in VTA neurons using DAR-4M-AM; *p* < 0.01 from control (*) or both control and MPP^+^ (**) groups by one-way ANOVA with Tukey’s *post hoc* test (*n* = 24-75 cells from eight independent experiments). Download Figure 6-2, TIF file.

The LTCC inhibitor (isradipine), RyR antagonists (DHBP and ruthenium red), NO synthase inhibitor (l-NAME) or an MCU inhibitor (Ru360) each prevented the MPP^+^-induced NO increase in SN neurons (Fig. 6*C*). The mechanism responsible for increased NO production in these cells therefore appears to involve Ca^2+^ entry through LTCC, followed by the activation of the RyR, and Ca^2+^-mediated activation of mitochondrial NOS (Fig. 2*A*). Additionally, DAT activity was unaffected by L-type channel blockade (Fig. 6*D*) and l-NAME was unable to prevent the buildup of Ca^2+^ in MPP^+^-treated SN neurons (Fig. 4*C*), suggesting that RyR nitrosylation and increased conductance (Kakizawa et al., 2012) did not mediate the increase of Ca^2+^ in toxin-exposed cells. Interestingly, neither of the above inhibitors were able to reduce MPP^+^-mediated increases in DAR-4M fluorescence in VTA neurons (Extended Data Fig. 6-2), suggesting that MPP^+^ leads to an increase in NO levels independently of Ca^2+^ in these cells.

### Rescue of neurons from MPP^+^-mediated mitochondria oxidation and toxicity

**Figure 7.**
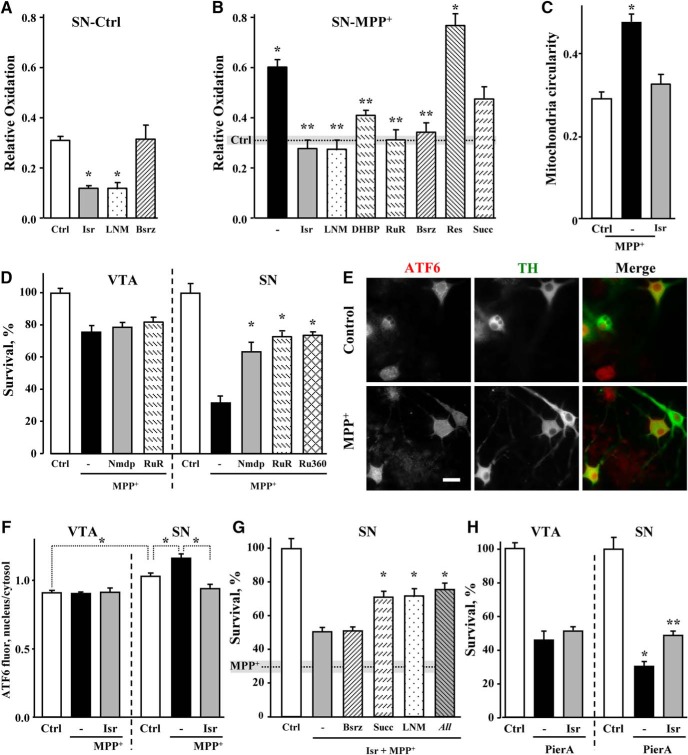
Rescue of DA neurons from MPP^+^-induced mitochondrial oxidation and toxicity. ***A***, ***B***, Relative oxidation of cultured SN neurons from TH-mito-roGFP mice in the absence (***A***) and in the presence (***B***) of 50 µM MPP^+^ for 2 h. Neurons were preincubated with metabolic effectors as indicated in the text and Figure 2*A*, legend; *p* < 0.001 from control (*) or MPP^+^ (**) by one-way ANOVA with Tukey’s *post hoc* test (*n* = 12-44 cells from 20 independent experiments). ***C***, Rescue of mitochondria morphology in SN neurons treated with 50 µM MPP^+^ for 2 h in the presence of 5 µM isradipine (30-min preincubation); *p < 0.05 from untreated cells by one-way ANOVA with Tukey’s *post hoc* test (*n* = 14-23 cells from three independent experiments). ***D***, MPP^+^-mediated toxicity in cultures pretreated with nimodipine (5 µM), ruthenium red (20 µM) or Ru360 (10 µM). None of the drugs were toxic to DA neurons in the absence of MPP^+^; *p < 0.001 from both control and MPP^+^ by one-way ANOVA with Tukey’s *post hoc* test (*n* = 3-23 dishes from seven independent experiments). ***E***, Representative images of SN neurons immunostained for TH and ATF6. Scale bar: 10 µm. ***F***, Quantification of the ratios of nuclear to cytosolic (perinuclear) ATF6 fluorescence in SN and VTA neurons treated with MPP^+^ for 24 h. Isradipine was added 30 min before MPP^+^; *p < 0.001 from control SN neurons by one-way ANOVA with Tukey’s *post hoc* test (*n* = 16-26 cells from two independent experiments). ***G***, Toxicity in SN cultures treated with both isradipine and MPP^+^. Dotted line and gray bar represent the level of survival (mean ± SEM) of SN neurons treated with MPP^+^ only. In the presence of the LTCC blocker, the sensitivity of SN neurons to different metabolic effectors became similar to that of VTA neurons (compare to Fig. 2*B*). Note, however, that none of the inhibitors combinations provided complete rescue of SN neurons from neurotoxicity. “*All*” designates a group pretreated with isradipine, Bsrz, succinate, and l-NAME simultaneously; *p < 0.01 from both control and MPP^+^ by one-way ANOVA with Tukey’s *post hoc* test (*n* = 6-28 dishes from 13 independent experiments). ***H***, Effect of the LTCC blockade on the survival of pierecidin A-treated SN and VTA neurons; *p* < 0.001 from control (*) or pierecidin A (**) by one-way ANOVA with Tukey’s *post hoc* test (*n* = 7-13 cells from four independent experiments).

We next investigated the contribution of DA_cyt_, Ca^2+^ and NO in mediating mitochondrial oxidative stress in naïve and MPP^+^-treated SN neurons. Both isradipine and l-NAME decreased mito-roGFP oxidation in control SN neurons (Fig. 7*A*), indicating that LTCC activity and downstream up-regulation of NOS are responsible for higher basal oxidative stress in these cells. Similarly, the same inhibitors as well as RyR antagonists (DHBP and ruthenium red), each decreased mitochondrial oxidation in MPP^+^-treated SN neurons (Fig. 7*B*). Furthermore, isradipine prevented MPP^+^-induced changes in mitochondrial morphology (Fig. 7*C*), highlighting the importance of Ca^2+^ in mediating the stress following toxin exposure. Blockade of DA synthesis with Bsrz had no effect on the oxidation of mitochondria in control SN neurons, but significantly decreased it in cells treated with MPP^+^, confirming a role for DA in mediating MPP^+^-induced oxidative stress (Fig. 7*A*,*B*). In contrast, the VMAT inhibitor reserpine had no significant effect, arguing against a possibility that DA released from synaptic vesicles is the source of oxidative stress in the soma of MPP^+^-treated neurons. Finally, restoring ETC function with succinate showed a trend for decreased mitochondrial oxidative stress in toxin-treated SN neurons, but the effect did not reach statistical significance (Fig. 7*B*).

As our data point to LTCC as important mediators of alterations in DA_cyt_ and NO in MPP^+^-treated neurons, we investigated whether pharmacological blockade of these channels is neuroprotective against the toxin, as reported for dihydropyridines *in vivo* (Chan et al., 2007). Nimodipine had no effect on the survival of VTA neurons exposed to MPP^+^, but provided a partial rescue to SN neurons, equalizing the difference in MPP^+^-induced toxicity between the two neuronal types (Fig. 7*D*). While DHBP was cytotoxic to midbrain cultures, blockade of RyR with ruthenium red or MCU with Ru360 each significantly increased the survival of SN but not VTA DA neurons (Fig. 7*D*).

To further investigate the mechanism of toxicity, we measured the level of ER stress by determining the intracellular localization of activating transcription factor 6 (ATF6). ER stress induced by Ca^2+^ store depletion leads to the proteolytic cleavage of ATF6 followed by the translocation of its cytosolic portion to the nucleus, where it acts as a transcription factor to upregulate expression of ER chaperones. We observed a significant increase in the nuclear to cytosolic ratio of ATF6 immunofluorescent label after 24 h of SN neurons exposure to MPP^+^ (Fig. 7*E*). Furthermore, in support of the observation that ER Ca^2+^ store depletion is secondary to LTCC opening, pretreatment with isradipine prevented MPP^+^-induced changes in ATF6 localization in SN but not VTA neurons (Fig. 7*F*).

Several experiments described above demonstrate that dihydropyridines counter changes in Ca^2+^, DA_cyt_ and NO in SN neurons, lowering the levels of these metabolites to those in VTA neurons. We therefore investigated if LTCC blockade also negates the difference in the contribution of various toxicity pathways in MPP^+^-treated SN and VTA neurons. Indeed, the difference in the neuroprotective potency of Bsrz, succinate and l-NAME in SN and VTA neurons (Fig. 2*B*) disappeared when SN neurons were pretreated with isradipine (Fig. 7*G*). We note that in contrast to complete neuroprotection of VTA neurons from MPP^+^ toxicity by succinate and l-NAME, there was only a partial rescue of isradipine-treated SN neurons with these inhibitors either alone or in combination. Thus, additional differences between the two neuronal populations, such as higher activity of DAT-mediated uptake of MPP^+^ in SN neurons (Fig. 1*D-F*) that was insensitive to dihydropyridine treatment (Fig. 6*D*), or the extensive arborization of cultured SN neurons (Pacelli et al., 2015) also played a role. Remarkably, isradipine also provided partial protection to SN, but not VTA neurons from piericidin A toxicity, demonstrating that Ca^2+^ influx via the LTCC is an important contributor to the sensitivity of SN neurons to mitochondrial stress such as Complex I inhibition (Fig. 7*H*).

### Role of aSyn in MPP^+^-mediated toxicity

**Figure 8.**
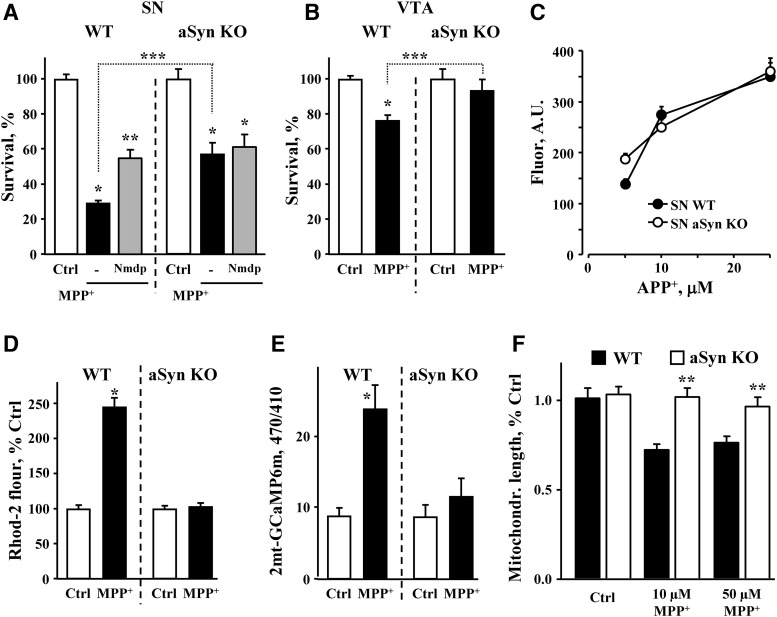
Rescue of MPP^+^-treated SN neurons by aSyn deletion. ***A***, Toxicity in SN cultures from WT and aSyn null mice treated with nimodipine and MPP^+^; *p* < 0.001 from corresponding control (*), both corresponding control and MPP^+^ (**) or WT and aSyn KO MPP^+^-treated groups (***) by one-way ANOVA with Tukey’s *post hoc* test (*n* = 6-16 dishes from three independent experiments). ***B***, Effect of aSyn deletion on MPP^+^-mediated toxicity in VTA DA cultures; *p* < 0.01 from corresponding control (*) or WT and aSyn KO MPP^+^-treated groups (***) by one-way ANOVA with Tukey’s *post hoc* test (*n* = 6-23 dishes from two independent experiments). ***C***, DAT-mediated uptake of APP^+^ is unchanged in aSyn-deficient SN neurons (*n* = 16-24 cells from two independent experiments). ***D***, MPP^+^ does not induce Ca^2+^ surge in SN neurons from aSyn null mice. WT and sSyn KO SN neurons were treated with 50 µM MPP^+^ for 2 h and changes in Ca^2+^ were accessed with Rhod2 (*n* = 41-124 neurons from five independent experiments). ***E***, Deletion of aSyn prevented the increase in mitochondrial Ca^2+^ in MPP^+^-treated SN neurons (*n* = 18-26 neurons from three independent experiments). ***F***, Mitochondria morphology did not change significantly in toxin-exposed SN neurons from aSyn null mice (*n* = 7-15 neurons from three independent experiments).

Finally, we examined whether expression of aSyn was required for selective MPP^+^ neurotoxicity. aSyn is a major component of Lewy bodies, a pathologic hallmark of PD, and is implicated in the pathology of the disease as mutations or overexpression of aSyn give rise to a dominant form of PD. Conversely, deletion of aSyn is protective in the MPTP mouse model of PD (Dauer et al., 2002), although the mechanism of neuroprotection is unknown. We found that, similar to *in vivo* reports, both SN and VTA neurons from aSyn-deficient mice (aSyn KO) were more resistant to MPP^+^-induced toxicity than neurons from WT animals (Fig. 8*A*,*B*).

Whereas DAT-mediated uptake (Fig. 8*C*) and DA_cyt_ levels (Mosharov et al., 2009) were unaffected by aSyn deletion, we observed no detectable Ca^2+^ increase in SN neurons following MPP^+^ treatment (Fig. 8*D*). Similarly, no effect of the toxin on mitochondrial Ca^2+^ (Fig. 8*E*) or morphology (Fig. 8*F*) were seen in aSyn KO neurons. These results suggest that the neuroprotection in the absence of aSyn involves a decrease in the MPP^+^-induced surge of Ca^2+^. Consistently, the effects of aSyn deletion and L-type channels blockade on neuronal survival were not additive (Fig. 8*A*).

**Figure 9.**
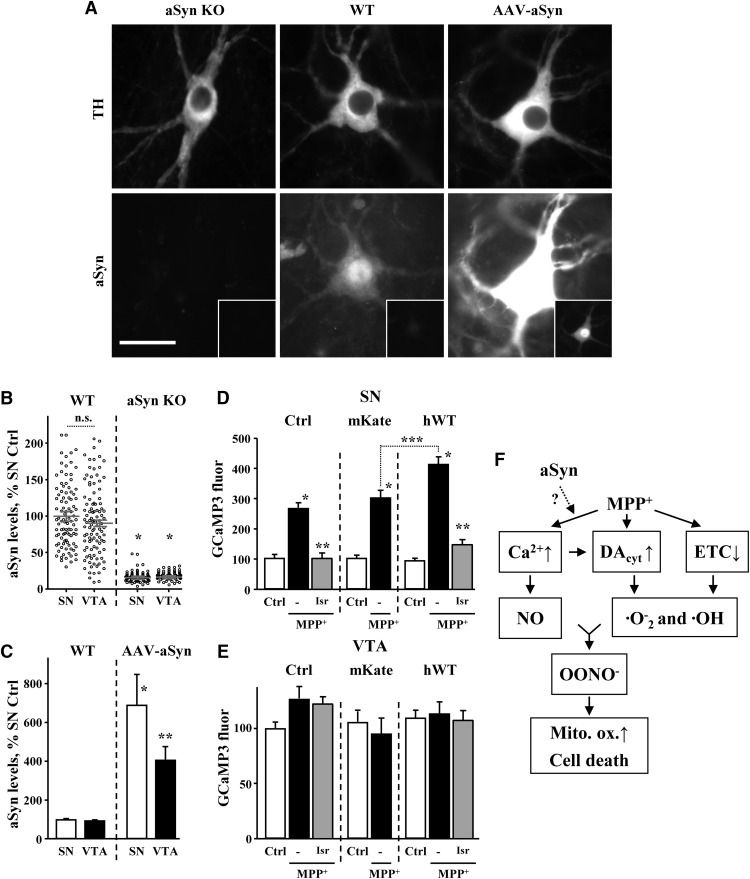
aSyn overexpression exacerbates the effect of MPP^+^ on Ca^2+^ in SN but not VTA neurons. ***A***, Representative images of WT, aSyn KO, and aSyn-overexpressing SN neurons immuostained against TH and aSyn with antibodies that react with both mouse and human protein. To overexpress aSyn, cultures were infected with AAV2 carrying human WT aSyn under the control of CBA promoter. Immunostaining was done 5 d after the infection. Insets show the same images at different brightness range to allow seeing the pattern of exogenous aSyn expression. Scale bar: 10 µm. Note that nuclear staining that is present in both WT and AAV-aSyn neurons is absent in aSyn KO neuorns. ***B***, Quantification of endogenous cytosolic aSyn immunolabel in cultured SN and VTA DA neurons; *p < 0.0001 from WT SN and VTA neurons (*n* = 83-127 neurons from two independent experiments). ***C***, Comparison of endogenous and exogenous aSyn levels in SN and VTA neurons treated with AAV2-haSyn; *p* < 0.01 from either WT (*) or both WT and SN AAV-aSyn groups (**) by one-way ANOVA with Tukey’s *post hoc* test (*n* = 38-104 cells from three independent experiments). ***D***, ***E***, Effect of human aSyn overexpression on cytosolic GCaMP3 fluorescence in SN (***D***) and VTA (***E***) DA neurons. One-week-old cultures of DAT-GCaMP3 neurons were infected with AAV2 carrying either mKate (red fluorescent tag) or human WT aSyn under the control of CBA promoter. GCaMP3 fluorescence in the presence and in the absence of MPP^+^ (50 µM MPP^+^ for 2 h) was monitored 5 d after the infection; *p* < 0.001 from corresponding control (*), corresponding MPP^+^ (**), or between indicated MPP^+^-treated groups (***) by one-way ANOVA with Tukey’s *post hoc* test (*n* = 20-75 cells for SN and 21-64 cells for VTA from four independent experiments). ***F***, Possible mechanism of neurotoxic interactions between Ca^2+^, DA_cyt_, and NO in MPP^+^-treated SN neurons.

As aSyn deletion provided such a strong effect on cell survival and Ca^2+^ levels in MPP^+^-treated SN neurons, we wondered if the reason that VTA neurons are more resistant to MPP^+^ is because of lower expression of aSyn. We thus compared aSyn levels in SN and VTA DA neurons by quantitative immunofluorescence. In WT DA neurons, we observed both cytosolic and nuclear aSyn staining that was absent in aSyn KO cultures (Fig. 9*A*), suggesting that aSyn may indeed be present in the nucleus as originally reported (Maroteaux et al., 1988). Comparison of fluorescence intensity in the cytosol of SN and VTA neurons from WT mice showed ∼10% higher aSyn expression in SN than VTA neurons (Fig. 9*B*), although the difference did not reach statistical significance.

We then addressed whether aSyn overexpression could potentiate MPP^+^-induced Ca^2+^ influx through the LTCC. We infected SN and VTA cultures from DAT-GCaMP3 mice either with an adeno-associated virus overexpressing human WT aSyn (hWT) or a red fluorescent protein mKate2 as control. Five days after infection, this produced robust overexpression of aSyn, reaching ∼5-fold increase in both SN and VTA neurons (Fig. 9*C*). While control or hWT aSyn viruses had no effect on GCaMP3 fluorescence in untreated cells, aSyn overexpression exacerbated the MPP^+^-induced Ca^2+^ increase in SN neurons (Fig. 9*D*). Furthermore, the toxin-induced Ca^2+^ increase was completely occluded by LTCC blockade with isradipine regardless of aSyn overexpression. In stark contrast to SN neurons, aSyn overexpression did not sensitize VTA neurons to MPP^+^, as GCaMP3 fluorescence was identical in untreated and MPP^+^-treated VTA neurons infected with either control or hWT viruses. Isradipine, similarly, had no effect on GCaMP3 fluorescence in VTA cultures (Fig. 9*E*).

## Discussion

Environmental neurotoxins such as MPTP and rotenone are widely used to induce selective degeneration of SN neurons *in vivo* and *in vitro* and mimic pathology in idiopathic PD, although the mechanisms responsible for this selectivity remain controversial (Hasbani et al., 2005; Jackson-Lewis and Smeyne, 2005; Choi et al., 2008). Perhaps the most prominent candidate for selective neurotoxicity has been higher DAT activity in SN neurons, but our data argue against this being the sole reason. First, while DAT-mediated uptake of MPP^+^ was indeed ∼40% greater in SN than VTA neurons, there were 60-70% higher levels and a different time course of toxicity in SN cultures (Fig. 1*A*,*B*). Second, the IC_50_ of nomifensine-mediated rescue of SN neurons from MPP^+^ was ∼8-fold higher than the IC_50_ of nomifensine-mediated blockade of DAT. Finally, pharmacological blockade of LTCC, which had no effect on DAT activity, abolished MPP^+^-induced metabolic differences between SN and VTA neurons and provided protection selectively to SN neurons. While the difference in DAT activity appears to play a minor role, we found that several key metabolic players, including Ca^2+^, DA, NO, and aSyn underlie the higher sensitivity of SN neurons to MPP^+^.

Ca_v_1.3 LTCC provide pacemaking activity in SN and LC neurons and it has been postulated that elevated cytoplasmic Ca^2+^ provides an energy load, leading to “wear and tear” of cellular homeostatic machinery (Surmeier, 2007). Furthermore, pharmacological blockade of the LTCC is neuroprotective against systemic MPTP administration *in vivo* (Kupsch et al., 1996; Chan et al., 2007). To investigate the role of Ca^2+^ in mediating the toxicity of MPP^+^, we employed several optical probes. Following the initial decrease in Ca^2+^ that was consistent with MPP^+^-mediated activation of K_ATP_ and Girk2 channels (Chung et al., 2005; Liss et al., 2005; Lüscher and Slesinger, 2010; Yee et al., 2014), we found that after 2-3h of exposure, cellular Ca^2+^ increased transiently and selectively in SN neurons by a mechanism that involved the activity of L-type channels and RyR, but not the IP_3_ receptors. As mitochondrial and cytoplasmic Ca^2+^ appeared to rise in parallel in toxin-treated neurons, the source of the Ca^2+^ surge likely involved an increase in plasma membrane or ER conductance, followed by mitochondrial uptake via MCU. Our data implicate the involvement of L-type channels during the initial increase in Ca^2+^, permitting the opening of the RyR to produce still higher toxin-induced Ca^2+^ elevation.

It is unknown whether and to what degree the membrane of DA neurons is permeable to the Complex II precursor succinate. It has, however, been recently demonstrated that cortical neurons express the Na^+^-coupled carboxylate transporter 2 and are able to accumulate [^14^C]succinate with an affinity and V_max_ of 7.3 ± 1.6 µM and 266.4 ± 15.2 pmol/mg protein/h, respectively (Yodoya et al., 2006). Here, we show that succinate did not prevent the buildup of Ca^2+^ and provided no rescue to MPP^+^-treated SN neurons, but enhanced the survival of VTA neurons (Fig. 2*B*) or isradipine-pretreated SN neurons (Fig. 7*G*), suggesting that ATP depletion was not the initial cause of biochemical changes that led to toxicity. Consistently, the glutathione precursor, NAC, rescued SN neurons (Fig. 2*B*) without affecting the MPP^+^-induced increase in Ca^2+^ (Fig. 4*C*), indicating that the Ca^2+^ increase preceded rather than followed cellular oxidative stress. Interestingly, selective Complex I blockade by piericidin A also produced higher toxicity in SN than VTA DA neurons (Fig. 2*C*) and, similar to MPP^+^ effects, this difference was negated by pretreatment with the LTCC inhibitor (Fig. 7*H*). This again suggests that ETC inhibition is an additive factor, rather than the source of all metabolic changes that follow.

The contribution of DA homeostasis to MPTP-induced toxicity has been controversial. Disruption of DA vesicular storage and inhibition of MAO by MPP^+^ lead to the accumulation of DA_cyt_, oxidative stress and cell death in midbrain cultures (Lotharius et al., 1999; Choi et al., 2015). However, SN neurons in animals that lack TH and therefore are unable to produce DA still undergo MPTP-induced degeneration (Hasbani et al., 2005). Our data suggest that the contribution of DA toxicity can differ even between closely related cell types. While there was no significant effect of DA depletion in MPP^+^-treated VTA neurons, DA-related stress played a role in mitochondrial oxidation and accounted for a large portion of MPP^+^ toxicity in SN neurons. Interestingly, LTCC basal activity, but not the MPP^+^-mediated increase in Ca^2+^, appears to underlie the different DA_cyt_ levels in SN and VTA neurons, as pharmacological or genetic blockade of the Ca_v_1.3 channels, but not of RyR, decreased DA_cyt_ in SN neurons, while the action of both channels was required for the toxin-induced increase in Ca^2+^. We hypothesize that while LTCC increased basal DA synthesis in SN neurons (Mosharov et al., 2009), the main reason for elevated DA_cyt_ in MPP^+^-treated neurons was the inhibition of MAO by the toxin (IC_50_ = 1 µM; Choi et al., 2015).

Our results confirm that inhibition of NOS (Schulz et al., 1995; Hantraye et al., 1996; Przedborski et al., 1996) is effective at countering toxin-induced mitochondria oxidation. Specifically, Ca^2+^-mediated induction of mitochondrial nNOS (isoform α; mtNOS; Traaseth et al., 2004; Marks et al., 2005) in SN neurons was responsible for their higher sensitivity to the toxin, as inhibition of mitochondrial Ca^2+^ uptake via MCU alleviated both the buildup of NO and toxicity in MPP^+^ -treated cells. Similar upregulation of Ca^2+^/NO with concomitant mitochondria oxidative stress was recently demonstrated in LC catecholaminergic neurons (Sanchez-Padilla et al., 2014) and SN neurons exposed to aSyn preformed fibrils (Dryanovski et al., 2013), indicating that this pathway may be commonly activated under stress conditions.aSyn is the best established participant in the molecular pathology of PD, as mutations in the SNCA gene or its multiplication produce monogenic forms of PD (Eriksen et al., 2005). Moreover, genome wide association studies found a link between the SNCA locus and the risk of sporadic PD (International Parkinson Disease Genomics Consortium et al., 2011). Participation of this protein in PD is further supported by the presence of aSyn in Lewy bodies (Spillantini et al., 1997), its increased expression and phosphorylation in postmortem brains of sporadic PD patients (Fujiwara et al., 2002; Anderson et al., 2006; Murphy et al., 2014) and an autoimmune response to the protein in PD patients (Sulzer et al., 2017). While it has long been recognized that aSyn-deficient DA neurons show increased resistance to MPTP/MPP^+^ toxicity (Dauer et al., 2002), the mechanism of neuroprotection remained elusive. Here, we demonstrate that in MPP^+^-treated SN neurons from aSyn KO mice, the pathologic surge of Ca^2+^ was not observed, preventing alterations in mitochondria morphology and cell death. In agreement with a recent study (Pathak et al., 2017), our data suggest that aSyn acts upstream of mitochondrial bioenergetics functions, most likely at the plasma membrane or ER where it modulates Ca^2+^ conductance. Interestingly, aSyn deletion was similar in effect to, and not additive with, the pharmacological blockade of LTCC that also nullified the differences between SN and VTA neurons and rescued SN neurons from MPP^+^ toxicity. The involvement of these two established players of PD pathogenesis on the same toxicity pathway provides a foundation for further studies of the relationship between the activity of LTCC in cells susceptible in PD, aSyn pathology, and RyR-mediated depletion of ER Ca^2+^ stores that induces the unfolded protein response.

Overexpression of aSyn has been reported to increase MPTP/MPP+-mediated toxicity both *in vitro* and *in vivo* (Lee et al., 2001; Chung et al., 2005; Nieto et al., 2006; Zhou et al., 2006; Qian et al., 2008; Zhu et al., 2012). We found that the effect of aSyn varied between cell types. In SN neurons, excess of aSyn exacerbated the increase in Ca^2+^ caused by MPP^+^ exposure, whereas in VTA neurons there was no detectable Ca^2+^ increase, regardless of aSyn expression levels (Fig. 9). These results demonstrate that (1) while aSyn expression is essential, it is not sufficient to elicit the MPP^+^-induced Ca^2+^ increase, which presumably required additional factors specifically expressed in SN neurons; (2) aSyn acts in concert with the LTCC; and (3) elevation of aSyn levels is insufficient to sensitize VTA neurons to MPP^+^.

Interestingly, while inhibitors of different toxicity pathways provided protection against MPP^+^, they were not more effective in combination. For example, isradipine and Bsrz each rescued SN neurons from MPP^+^-induced degeneration, but their effect was not additive (Figs. 2*B*, 7*G* ). Similarly, l-NAME and succinate each decreased MPP^+^ toxicity in isradipine-treated SN neurons, but the effect of both drugs applied simultaneously was the same as for each applied independently. Perhaps most surprisingly, the MPP^+^-induced oxidation of mitochondria was rescued with equal potency by isradipine, l-NAME, DHBP, RuR, and Bsrz, indicating that removing any of the stressors was sufficient to alleviate the toxicity (Fig. 7*B*). Thus, neurotoxicity may result from the synergistic interaction between several toxicity pathways in SN neurons. For example, generation of superoxide and hydroxyl radicals due to ETC inhibition and accumulation of DA_cyt_ (Fridovich, 1983; Fahn and Cohen, 1992) combined with Ca^2+^-mediated upregulation of NO synthesis may give rise to reactive nitrogen species, such as peroxynitrite (Przedborski et al., 1996; Pennathur et al., 1999; Fig. 9*F*). Alternatively, cellular redox defense systems might be overwhelmed when levels of Ca^2+^, DA_cyt_, and NO increase simultaneously, but are able to cope with the stress when one of the metabolites’ concentration is normalized; further work is required to distinguish between these and other possibilities. Several other questions still remain, including the reason for the difference in the time course of cell death in SN and VTA neurons, involvement of specific plasma membrane, ER and mitochondrial Ca^2+^ channels, and the effect of pathogenic mutant aSyn on Ca^2+^ homeostasis and toxicity in this toxicity model. Most importantly, the specifics of the mechanism by which MPP^+^ induces Ca^2+^ surge in SN neurons needs further investigation.

Finally, we note that our results may have applicability beyond understanding the mechanisms of MPP^+^-induced cell death. Our findings with this parkinsonian neurotoxin, are mechanistically similar to a recent study of DA- and aSyn-mediated toxicity in human idiopathic and familial IPSC-derived DA neurons and of DJ-1 (PARK7) deficient mouse SN neurons *in vivo* (Burbulla et al., 2017). MPP^+^ thus mimics several of the cardinal pathologic features of idiopathic and genetic PD, allowing us to unmask a novel interaction between aSyn, L-type calcium channels, and mitochondrial ROS which provides a blueprint through which genetic forms of PD caused by pathogenic aSyn may interface with sporadic forms related to elevated calcium burden and mitochondrial dysfunction. Our results buttress the hypothesis that selective degeneration of SN neurons in PD results from multiple genetic and neurotoxic “hits” that conspire to overwhelm cellular defense mechanisms (Sulzer, 2007; Mosharov et al., 2009).

**Table 1:** Details of statistical analyses between all experimental groups presented in the paper.

Figure	Test used	*n*	Defined	*p* value	*F*/*t* value
1*A*	Two-way ANOVA	VTA: 23, 13, 15, 14; SN-16, 9, 12, 10	Dishes from 12 independent experiments	*p* < 0.0001	*F*_(1,104)_ = 412.4
1*B*	Two-way ANOVA	VTA: 3, 5, 5, 3, 3; SN- 5, 4, 2, 6, 3	Cultures from 2 independent experiments	*p* < 0.0001	*F*_(1,29)_ = 109.5
1*E*	Unpaired *t* test	4, 4	Cells in each group	*p* = 0.0294	*t*_(6)_ = 2.84
2*B*, VTA	One-way ANOVA	16, 8, 6, 7, 6, 6	VTA dishes from 8 independent experiments	*p* < 0.0001	*F*_(5,41)_ = 15.5
2*B*, SN	One-way ANOVA	17, 16, 7, 8, 10, 9	SN dishes from 9 independent experiments	*p* < 0.0001	*F*_(5,61)_ = 93.56
2*C*	Two-way ANOVA	VTA: 16, 8, 15, 6, 13; SN-11, 6, 10, 5	Dishes from 6 independent experiments	*p* < 0.001	*F*_(1,69)_ = 12.0
2*E*	One-way ANOVA	14, 17, 26, 23	Cells from 2 independent experiments	*p* < 0.0001	*F*_(3,76)_ = 14.47
2*G*	One-way ANOVA	40, 29, 106, 75	Cells from 14 independent experiments	*p* < 0.0001	*F*_(3,246)_ = 78.69
2*H*	Two-way ANOVA	VTA: 40, 27, 25, 30; SN: 70, 54, 22, 23	Cells from 11 independent experiments	*p* < 0.0001	*F*_(1,283)_ = 206.7
3*A*	Two-way ANOVA	VTA: 33, 6, 29, 16, 11, 19, 33, 7, 10, 19, 11, 15; SN: 34, 16, 18, 17, 11, 16, 34, 8, 19, 24, 18, 12	Cells from 8 independent experiments	*p* < 0.0001	*F*_(3,412)_ = 43.15
3*B*	Unpaired *t* test	6, 8	Cells from 2 independent experiments	*p* < 0.05	*t*_(12)_ = 3.52
3*D*	Two-way ANOVA	VTA: 71, 27, 38, 11; SN: 57, 24, 35, 23	Cells from 7 independent experiments	*p* < 0.0001	*F*_(1,278)_ = 73.09
4*B*	Two-way ANOVA	VTA: 23, 14, 24, 24; SN: 51, 36, 22, 9	Cells from 4 independent experiments	*p* < 0.0001	*F*_(1,195)_ = 101.1
4*C*	One-way ANOVA	VTA: 46, 23, 51, 35; SN: 68, 11, 100, 59, 92, 48, 29, 41, 43	Cells from 14 independent experiments	*p* < 0.0001	*F*_(12,633)_ = 60.75
4*D*	One-way ANOVA	VTA: 67, 37; SN: 68, 48, 37	Cells from 3 independent experiments	*p* < 0.0001	*F*_(4,192)_ = 9.16
5*C*	One-way ANOVA	VTA: 36, 57; SN: 75, 46, 54, 20, 42, 28, 28	Cells from 8 independent experiments	*p* < 0.0001	*F*_(8,377)_ = 52.62
5*E*	One-way ANOVA	VTA: 24, 28; SN: 18, 26, 29	Cells from 3 independent experiments	*p* < 0.0001	*F*_(4,120)_ = 9.32
6*A*	One-way ANOVA	VTA: 9, 10, 10, 9; SN: 25, 28, 14, 9, 10, 10	Cells from 4 independent experiments	*p* < 0.0001	*F*_(9,124)_ = 40.1
6*B*	One-way ANOVA	VTA: 64, 42, 25; SN: 54, 38, 20	Cells from 7 independent experiments	*p* < 0.0001	*F*_(5,127)_ = 31.04
6*C*	One-way ANOVA	80, 26, 15, 21, 78, 33, 29, 43, 22, 21	Cells from 14 independent experiments	*p* < 0.0001	*F*_(9,358)_ = 20.75
6*D*	Two-way ANOVA	16, 20, 18, 16	Cells from 2 independent experiments	*p* = 0.4593	*F*_(1,66)_ = 0.55
7*A*	One-way ANOVA	24, 15, 22, 12	Cells from 5 independent experiments	*p* < 0.0001	*F*_(3,246)_ = 78.69
7*B*	One-way ANOVA	24, 44, 25, 17, 38, 27, 33, 9, 19	Cells from 15 independent experiments	*p* < 0.0001	*F*_(8,227)_ = 13.69
7*C*	One-way ANOVA	14, 17, 23	Cells from 3 independent experiments	*p* < 0.0001	*F*_(2,51)_ = 19.0
7*D*, VTA	One-way ANOVA	16, 10, 5, 3	Dishes from 5 independent experiments	*p* < 0.0001	*F*_(3,30)_ = 12.28
7*D*, SN	One-way ANOVA	17, 23, 8, 8, 6	Dishes from 7 independent experiments	*p* < 0.0001	*F*_(4,57)_ = 139.2
7*F*	One-way ANOVA	VTA: 26, 23, 22; SN: 16, 23, 22	Cells from 2 independent experiments	*p* < 0.05	*F*_(5,126)_ = 13.89
7*G*	One-way ANOVA	22, 28, 25, 10, 6, 6, 10	Dishes from 13 independent experiments	*p* < 0.0001	*F*_(6,99)_ = 157.6
7*H*	One-way ANOVA	VTA: 12, 12, 13; SN: 7, 7, 8	Dishes from 4 independent experiments	*p* < 0.0001	*F*_(5,53)_ = 95.53
8*A*	One-way ANOVA	WT: 16, 12, 8; KO: 14, 11, 6	Dishes from 3 independent experiments	*p* < 0.0001	*F*_(5,58)_ = 80.15
8*B*	One-way ANOVA	23, 15, 6, 6	Dishes from 2 independent experiments	*p* < 0.01	*F*_(3,46)_ = 14.12
8*C*	Two-way ANOVA	WT: 18, 20, 18; KO: 20, 16, 24	Cells from 2 independent experiments	*p* = 0.4988	*F*_(1,110)_ = 0.46
8*D*	One-way ANOVA	104, 124, 41, 83	Cells from 5 independent experiments	*p* < 0.0001	*F*_(3,348)_ = 59.46
8*E*	One-way ANOVA	18, 26, 18, 19	Cells from 3 independent experiments	*p* < 0.0001	*F*_(3,77)_ = 8.15
8*F*	Two-way ANOVA	WT: 7, 10, 15; KO: 7, 10, 10	Cells from 3 independent experiments	*p* < 0.0001	*F*_(1,53)_ = 20.67
9*B*	One-way ANOVA	97, 104, 127, 83	Cells from 2 independent experiments	*p* < 0.0001	*F*_(3,407)_ = 224.8
9*C*	One-way ANOVA	97, 104, 38, 41	Cells from 3 independent experiments	*p* < 0.0001	*F*_(3,276)_ = 32.04
9*D*	One-way ANOVA	Ctrl: 75, 54, 20; mKate: 40, 42; hWT: 50, 52, 54	Cells from 4 independent experiments	*p* < 0.0001	*F*_(7,379)_ = 63.87
9*E*	One-way ANOVA	Ctrl: 36, 57, 61; mKate: 21, 21; hWT: 64, 64, 33	Cells from 4 independent experiments	*p* = 0.7016	*F*_(7,349)_ = 0.67
Extended Data figures					
1-1*C*	Two-way ANOVA	VTA:13; SN: 11	Cells in 2 independent experiments	*p* < 0.01	*F*_(1,392)_ = 9.85
1-1*D*	Mann-Whitney	13, 11	Cells in 2 independent experiments	*p* = 0.506	*U* = 60.5
1-1*E*	Two-way ANOVA	VTA: 13; SN: 11	Cells in 2 independent experiments	*p* = 0.053	*F*_(1,175)_ = 3.79
1-1*F*	Mann-Whitney	15, 11	Cells in 2 independent experiments	*p* = 0.643	*U* = 80.0
1-1*G*	Mann-Whitney	14, 12	Cells in 2 independent experiments	*p* = 0.699	*U* = 76.0
1-1*H*	Mann-Whitney	14, 12	Cells in 2 independent experiments	*p* = 0.625	*U* = 74.0
1-2*A*	Two-way ANOVA	WT: 16, 12, 10; THGFP: 6, 5, 5, 5; THmito: 6, 5, 5, 5; DATGCaMP: 6, 5, 5, 5	Cells from 6 independent experiments	*p* = 0.4232	*F*_(3,74)_ = 0.95
1-2*B*	Two-way ANOVA	WT: 23, 15, 14; THGFP: 6, 5, 5, 5; THmito: 6, 5, 6, 5; DATGCaMP: 6, 5, 5, 5	Cells from 6 independent experiments	*p* = 0.9491	*F*_(3,89)_ = 0.12
4-1	Unpaired *t* test	33, 32	Cells from 2 independent experiments	*p* = 0.8678	*t*_(63)_ = 0.1675
5-1*B*	Two-way ANOVA	VTA: 18, 8, 26; SN: 34, 7, 37; effect of the drug	Cells from 4 independent experiments	*p* < 0.002	*F*_(2,131)_ = 6.61
5-1*B*	Two-way ANOVA	VTA: 18, 8, 26; SN: 34, 7, 37; effect between the cell types	Cells from 4 independent experiments	*p* = 0.1429	*F*_(1,131)_ = 2.17
5-1*C*	One-way ANOVA	36, 24, 57, 61	Cells from 7 independent experiments	*p* = 0.1601	*F*_(3,174)_ = 1.74
5-2	One-way ANOVA	68, 100, 59, 28, 29, 14	Cells from 7 independent experiments	*p* < 0.0001	*F*_(5,292)_ = 37.02
6-1	One-way ANOVA	33, 25, 9, 27, 30	Cells from 6 independent experiments	*p* < 0.0001	*F*_(4,119)_ = 9.47
6-2	One-way ANOVA	74, 27, 75, 33, 24, 40	Cells from 8 independent experiments	*p* < 0.0001	*F*_(5,267)_ = 26.46

## Synthesis

Reviewing Editor: Jörg Schulz, RWTH Aachen University Decisions are customarily a result of the Reviewing Editor and the peer reviewers coming together and discussing their recommendations until a consensus is reached. When revisions are invited, a fact-based synthesis statement explaining their decision and outlining what is needed to prepare a revision will be listed below.

I agree with one of the reviewers stating that their might still be some methodological issues in response to point 9/10. On the other hand, the culture system is well characterized and has frequently used in the the past. I would like to ask the authors to add a brief discussion on the possibility of “dedifferentiation” so that the comment of the reviewer is taken into account in the manuscript.

Alos point 5 is a valid critique. Here, I agree with the authors that the requested experiments are beyond the current scope of the manuscript and would take too long to be included. I would like to see the results published earlier. The authors discussed this point sufficiently in the manuscript.

Unfortunately, changes in the revised ms were not highlighted, making it less easy to evaluate the changes / improvements made to the ms.
