# Supplementary figures and images for: α-Synuclein-Dependent Calcium Entry Underlies Differential Sensitivity of Cultured SN and VTA Dopaminergic Neurons to a Parkinsonian Neurotoxin
Source: eNeuro. 2017 Nov 21;4(6):ENEURO.0167-17.2017. doi: 10.1523/ENEURO.0167-17.2017 (PMC5701296; doi:10.1523/ENEURO.0167-17.2017)

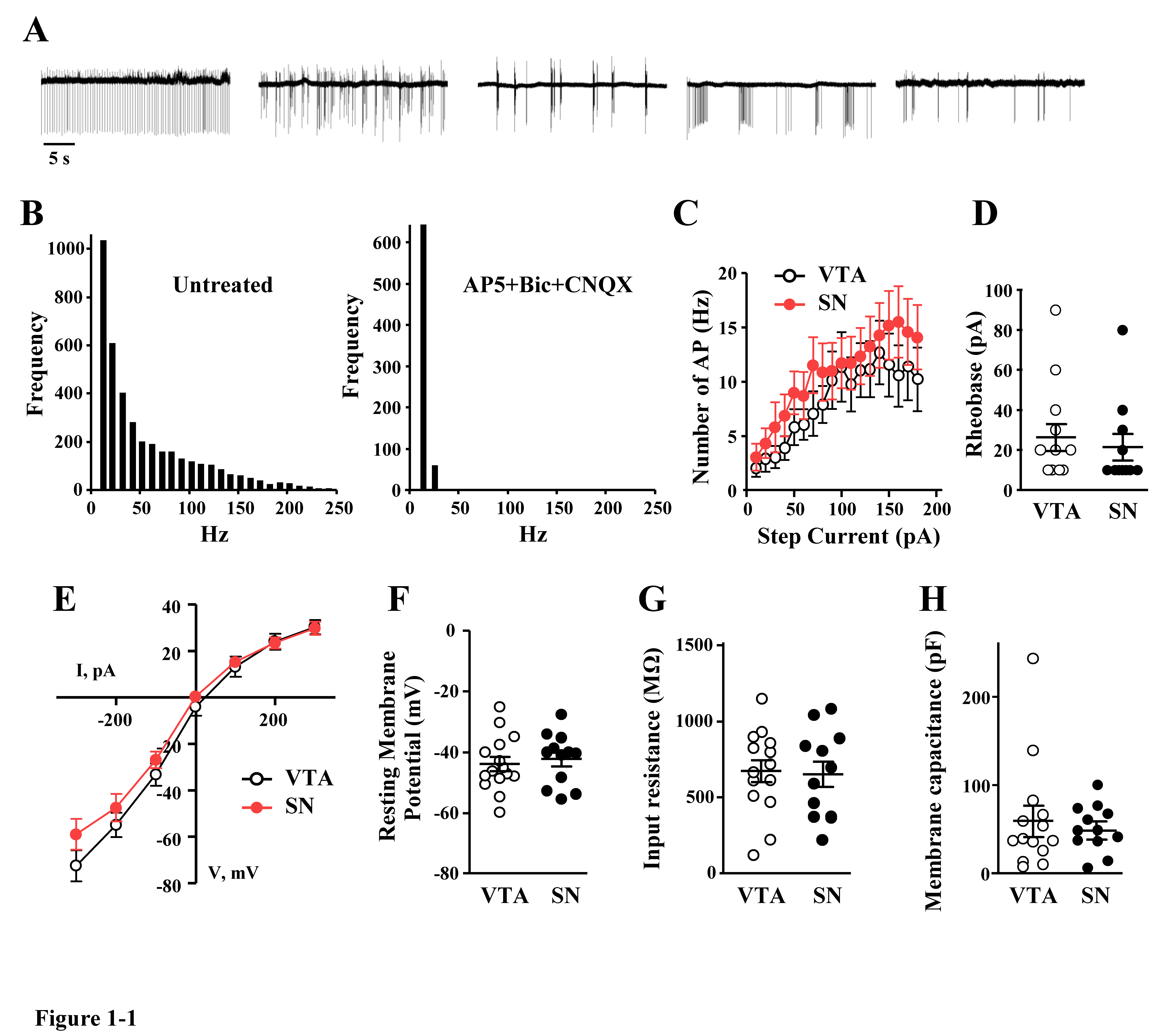

Supplement: Figure 1-1 [file enu006172457so10.tif]

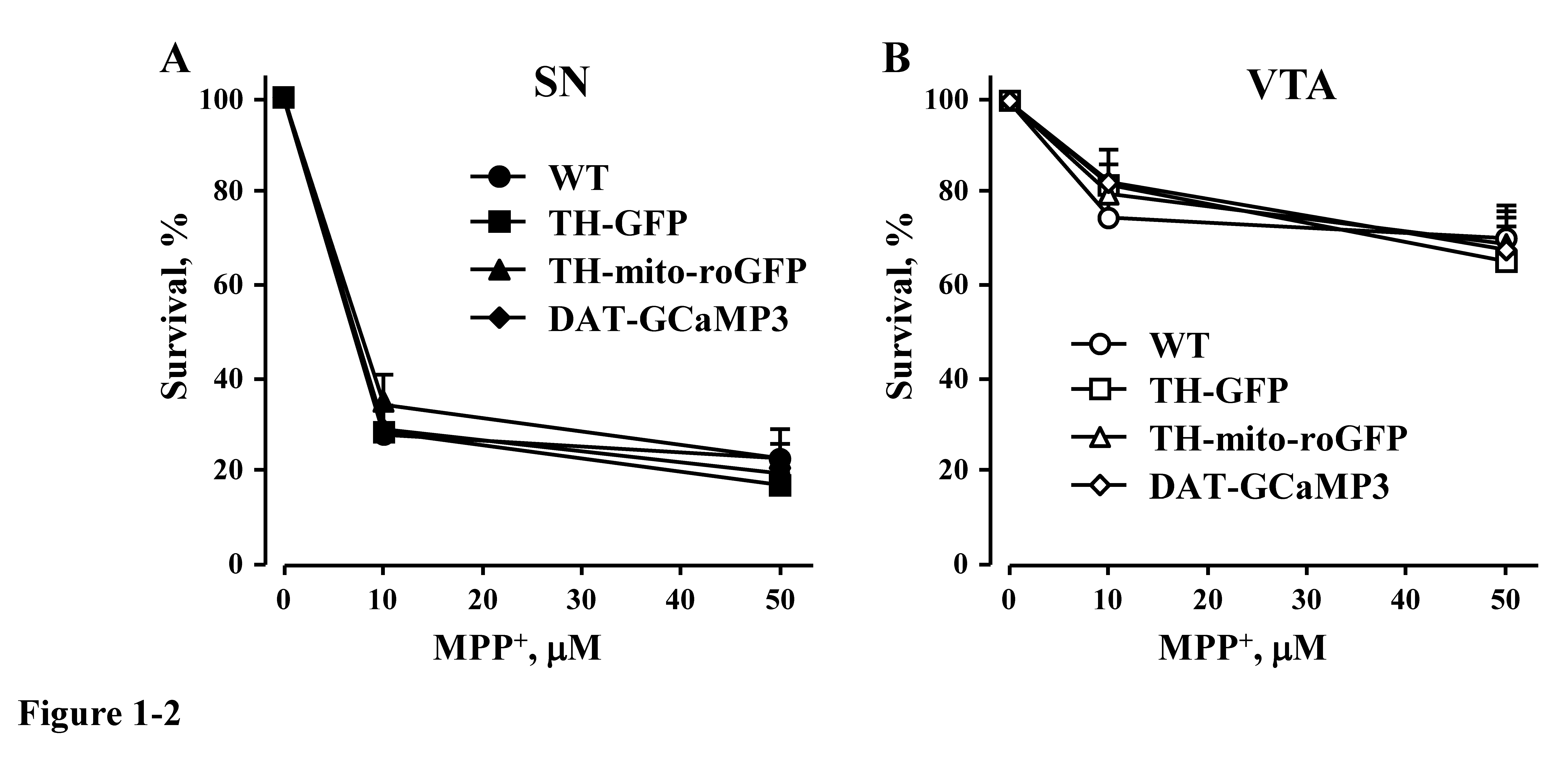

Supplement: Figure 1-2 [file enu006172457so11.tif]

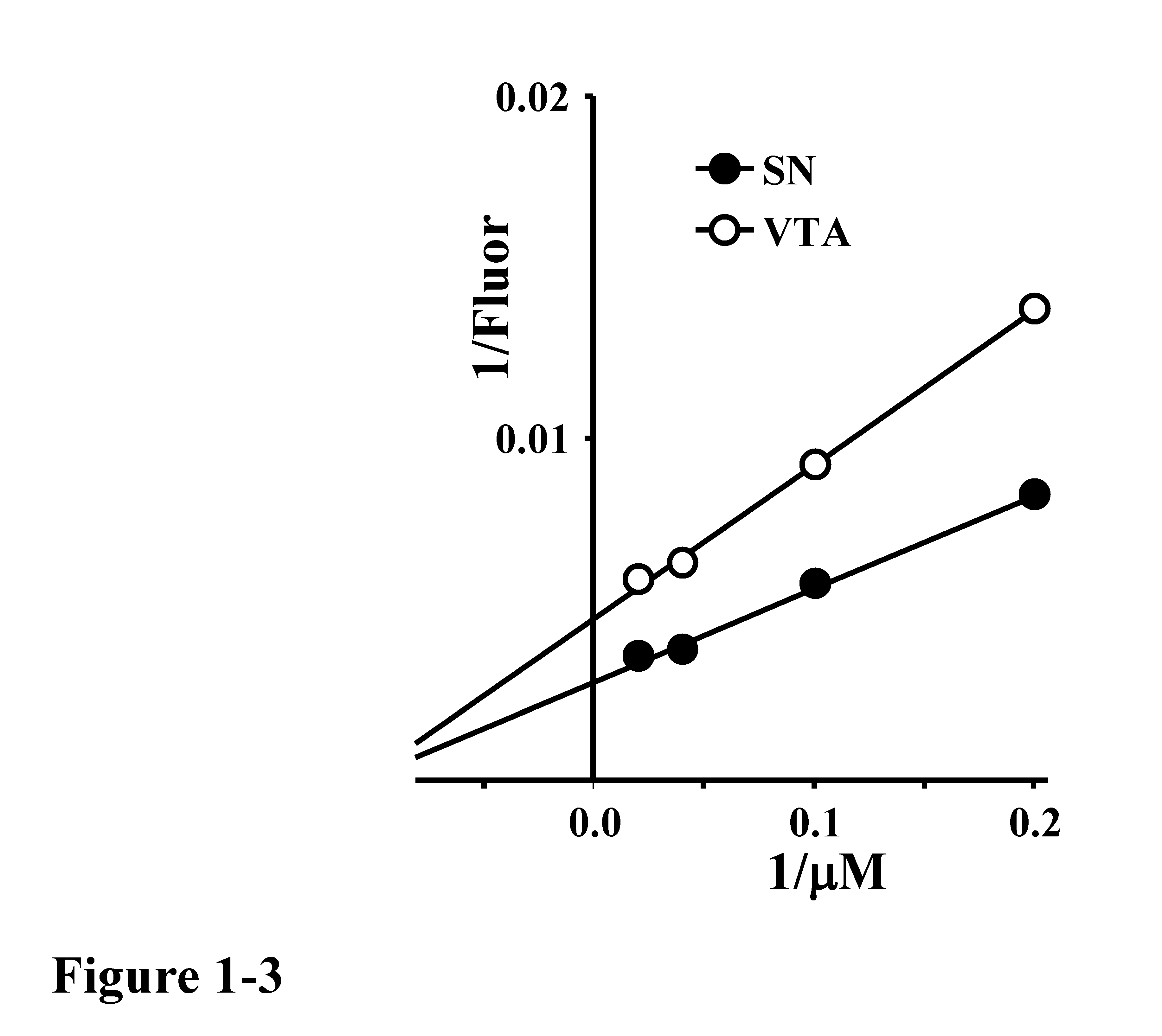

Supplement: Figure 1-3 [file enu006172457so12.tif]

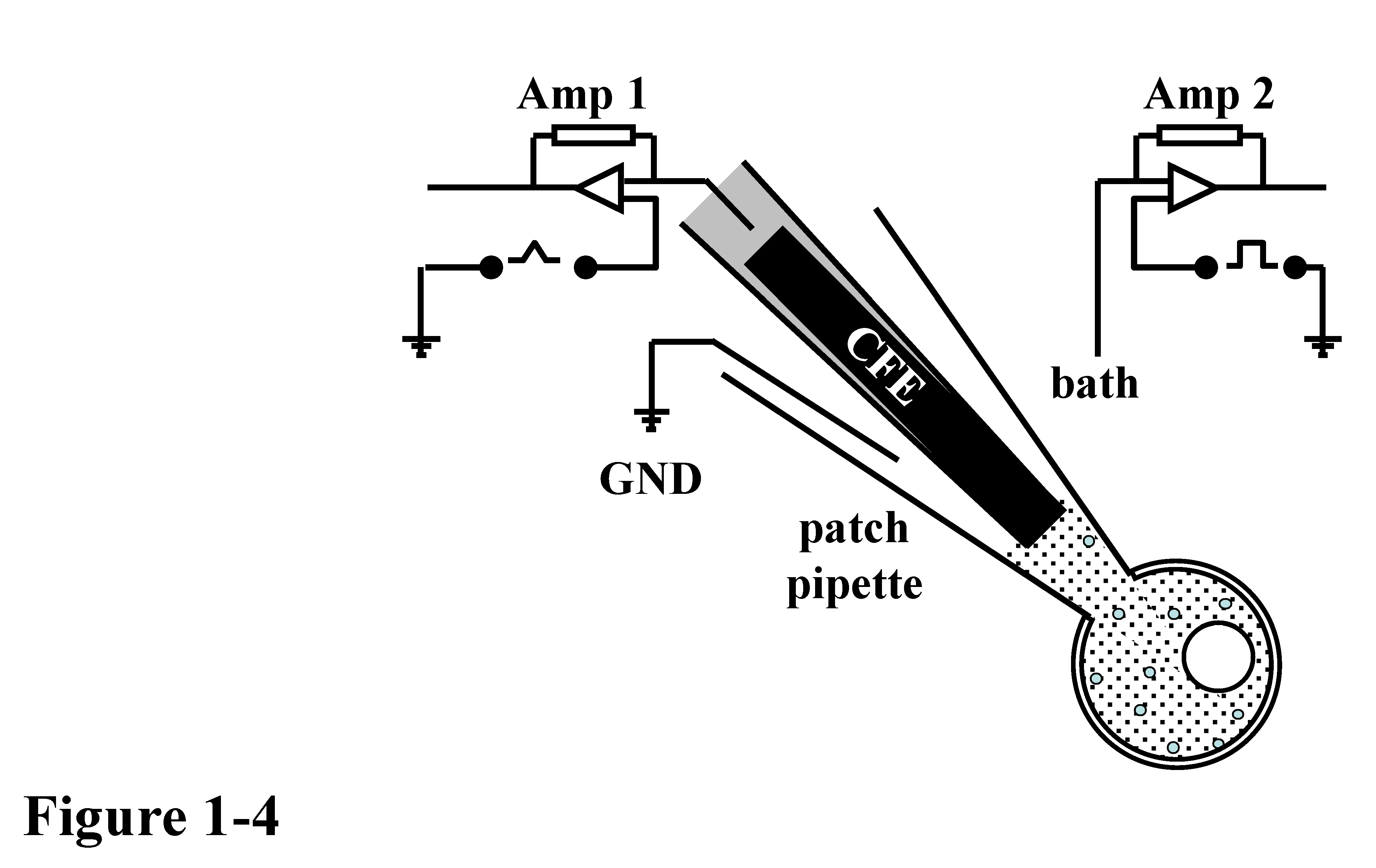

Supplement: Figure 1-4 [file enu006172457so13.tif]

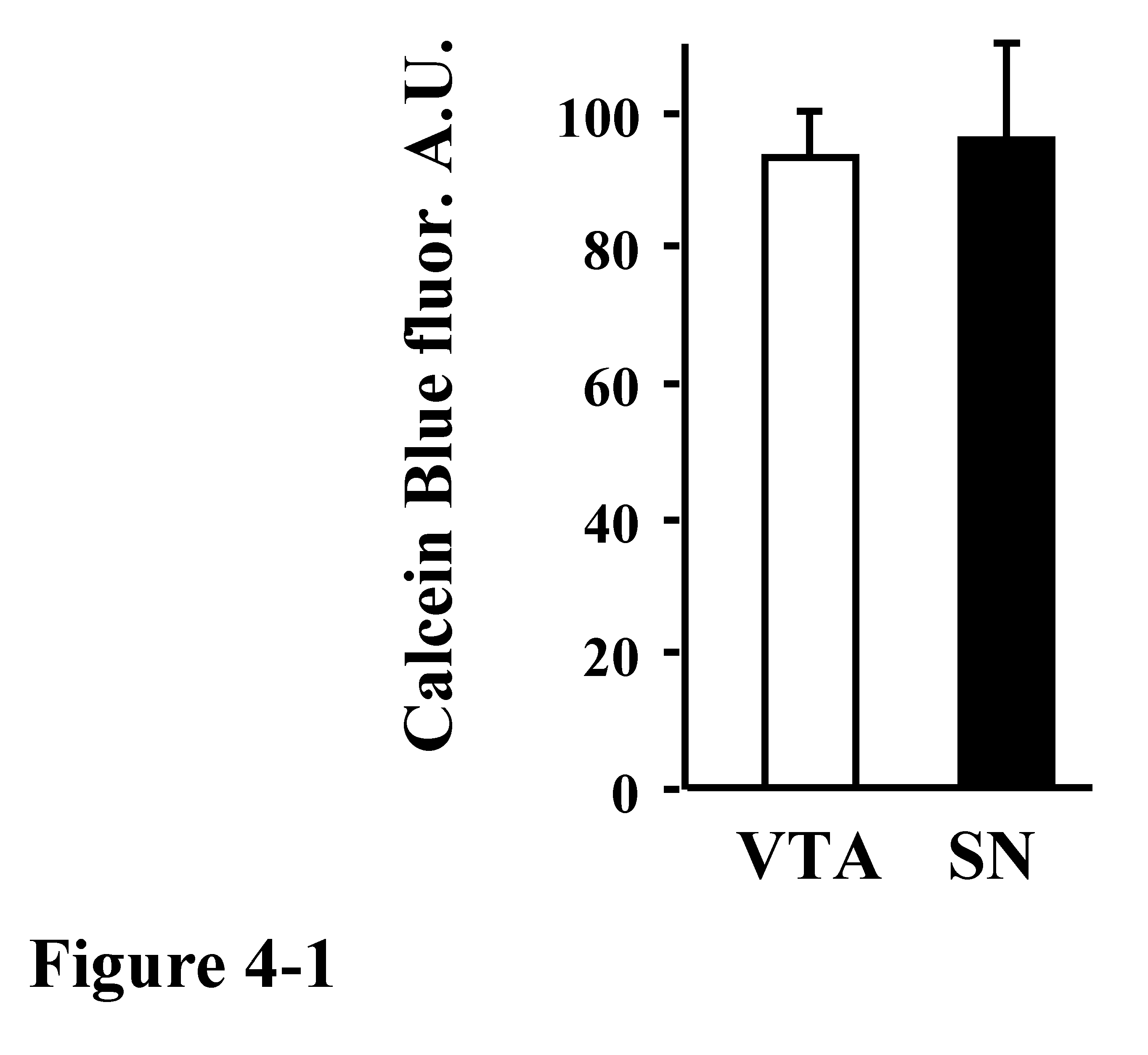

Supplement: Figure 4-1 [file enu006172457so14.tif]

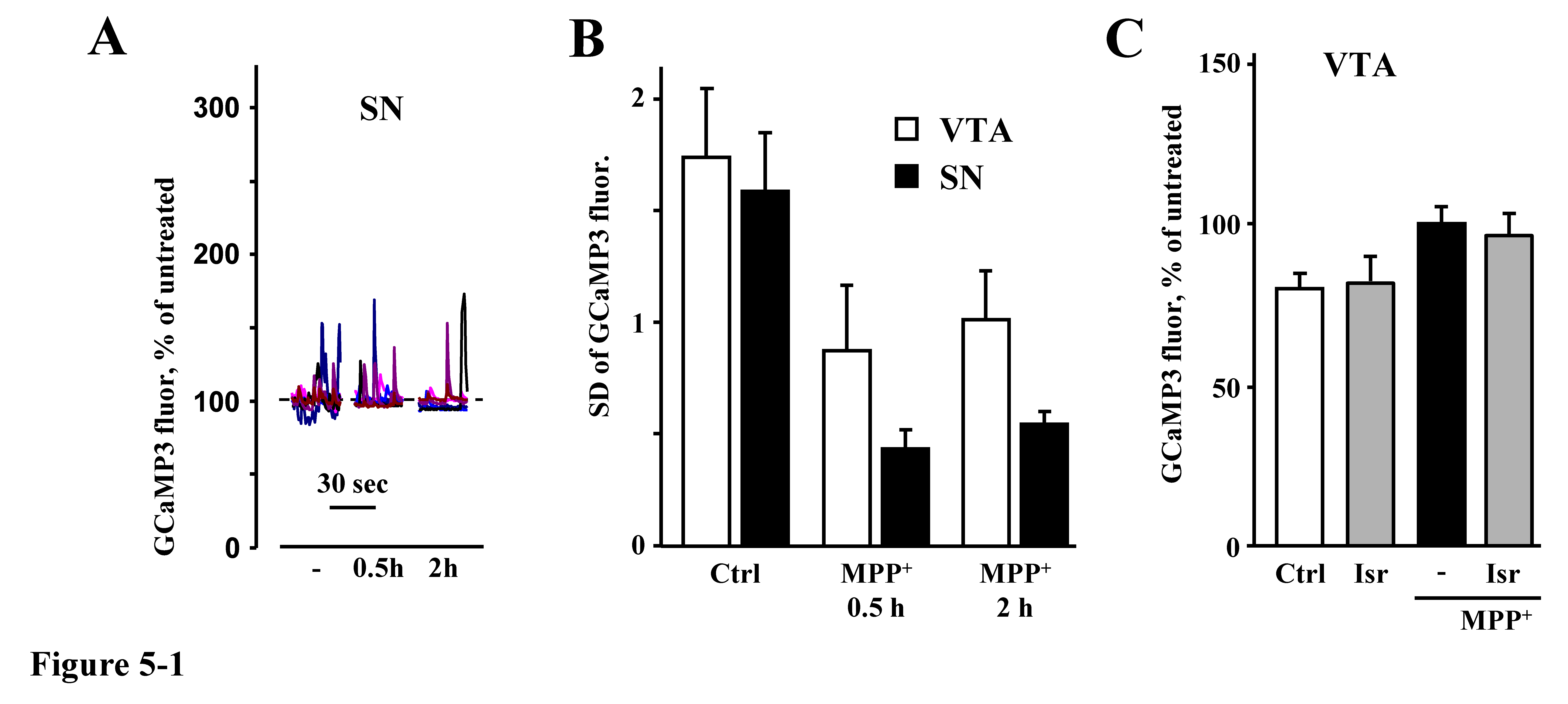

Supplement: Figure 5-1 [file enu006172457so15.tif]

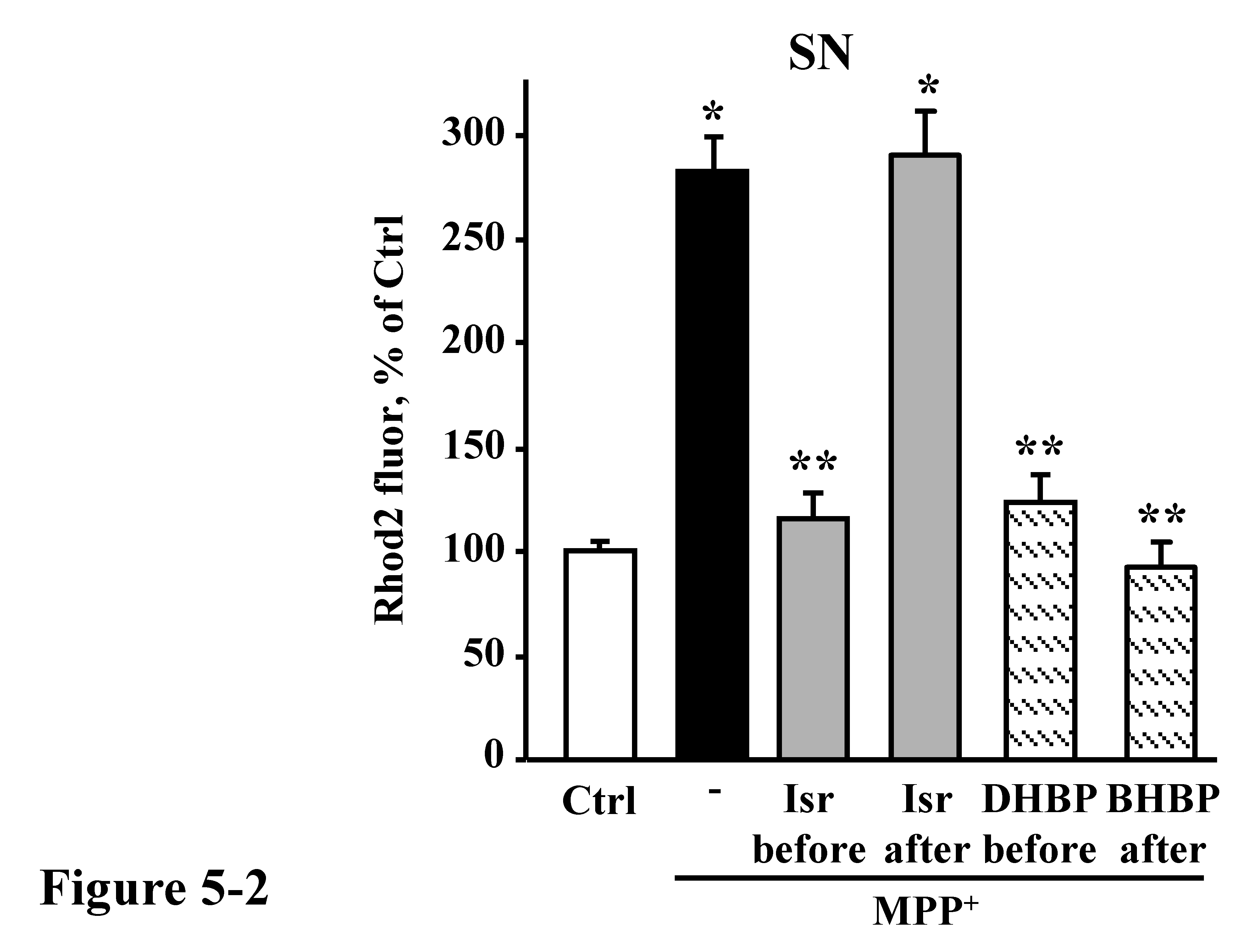

Supplement: Figure 5-2 [file enu006172457so16.tif]

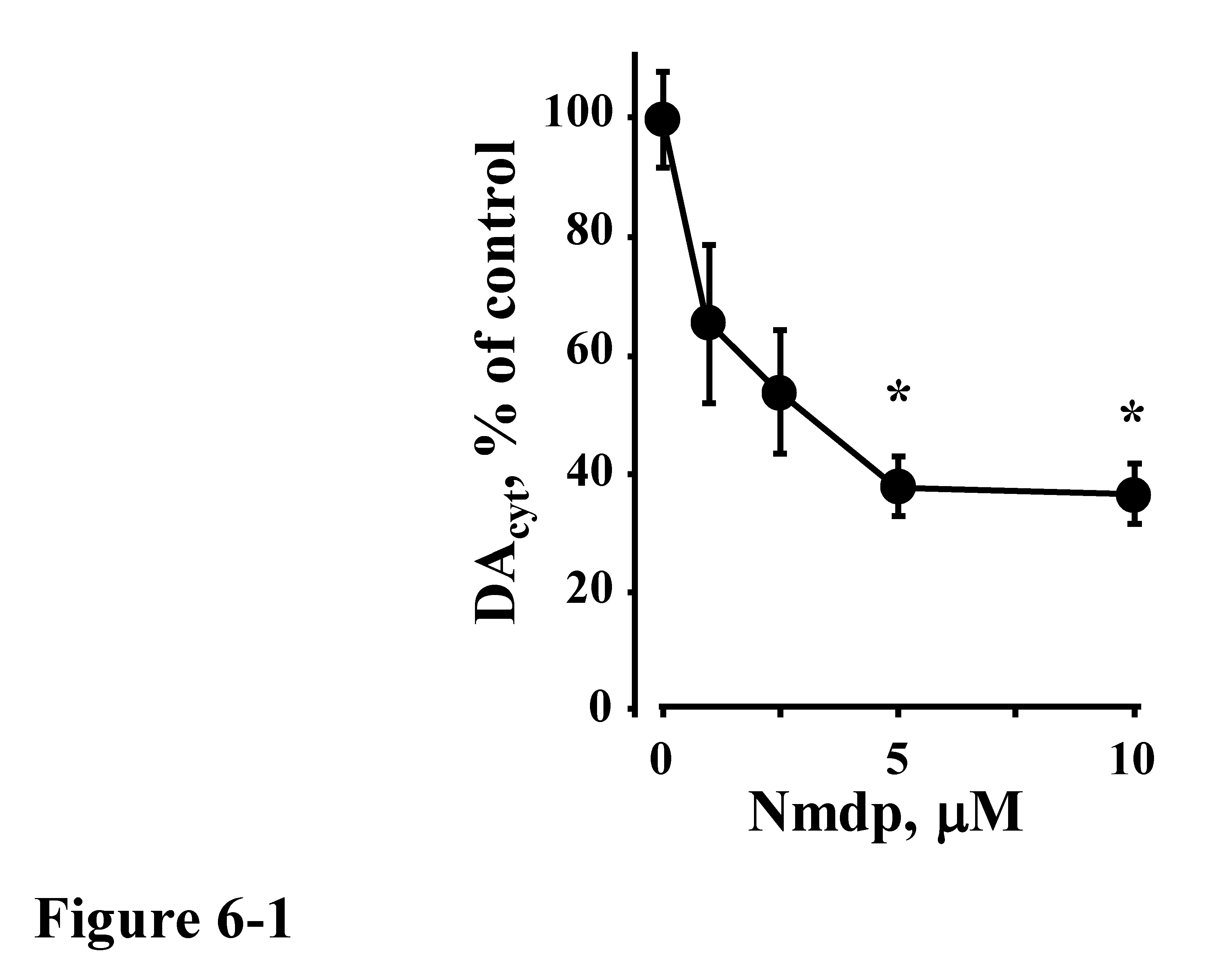

Supplement: Figure 6-1 [file enu006172457so17.tif]

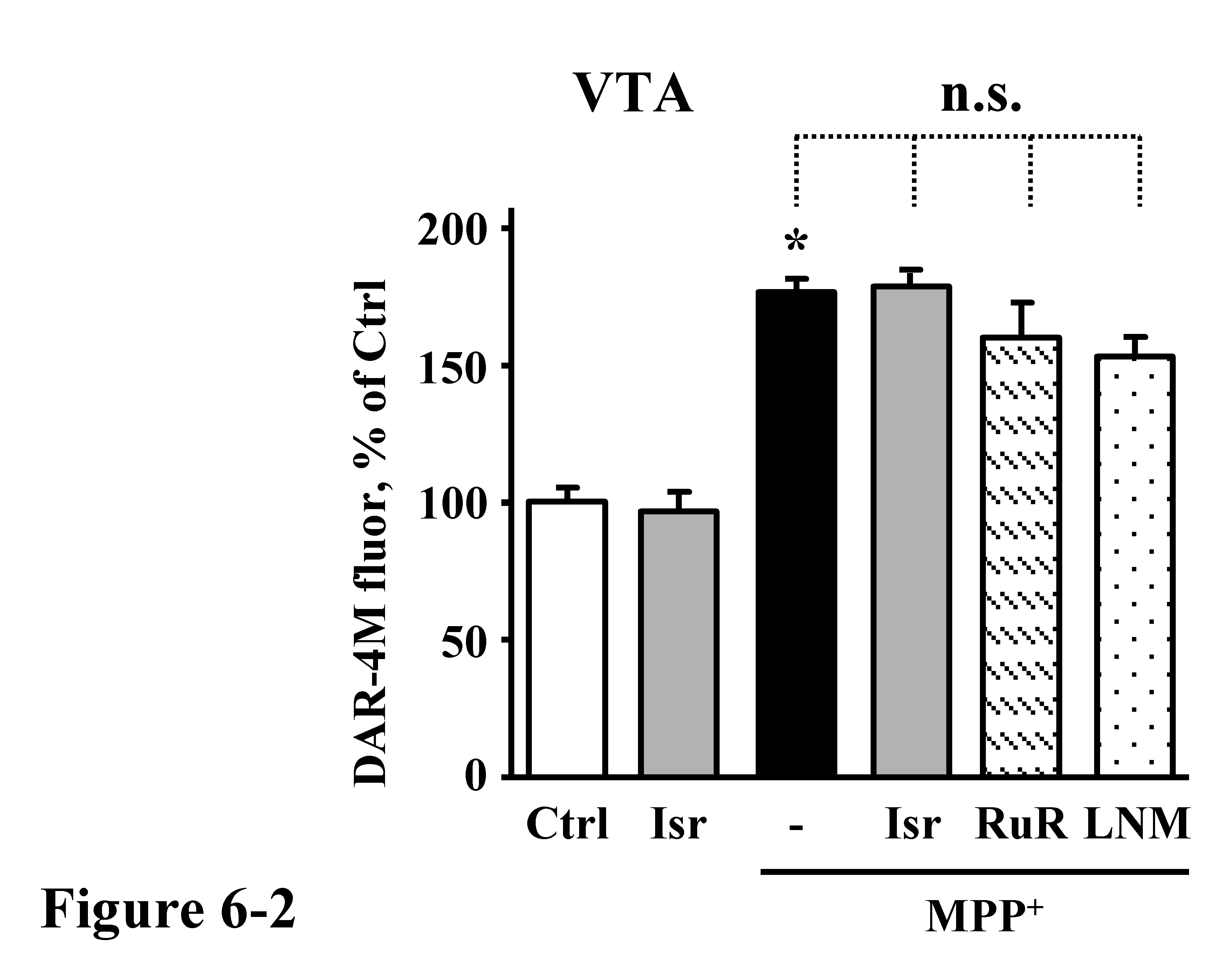

Supplement: Figure 6-2 [file enu006172457so18.tif]
